# Lamiaceae: An Insight on Their Anti-Allergic Potential and Its Mechanisms of Action

**DOI:** 10.3389/fphar.2019.00677

**Published:** 2019-06-19

**Authors:** Lee Yen Sim, Nur Zahirah Abd Rani, Khairana Husain

**Affiliations:** Drug and Herbal Research Centre, Faculty of Pharmacy, Universiti Kebangsaan Malaysia, Kuala Lumpur, Malaysia

**Keywords:** Lamiaceae, anti-allergic, allergy, hypersensitivity, mast cell, β-hexosaminidase, eosinophil, histamine

## Abstract

The prevalence of allergic diseases such as asthma, allergic rhinitis, food allergy and atopic dermatitis has increased dramatically in recent decades. Conventional therapies for allergy can induce undesirable effects and hence patients tend to seek alternative therapies like natural compounds. Considering the fact above, there is an urgency to discover potential medicinal plants as future candidates in the development of novel anti-allergic therapeutic agents. The Lamiaceae family, or mint family, is a diverse plant family which encompasses more than 7,000 species and with a cosmopolitan distribution. A number of species from this family has been widely employed as ethnomedicine against allergic inflammatory skin diseases and allergic asthma in traditional practices. Phytochemical analysis of the Lamiaceae family has reported the presence of flavonoids, flavones, flavanones, flavonoid glycosides, monoterpenes, diterpenes, triterpenoids, essential oil and fatty acids. Numerous investigations have highlighted the anti-allergic activities of Lamiaceae species with their active principles and crude extracts. Henceforth, this review has the ultimate aim of compiling the up-to-date (2018) findings of published scientific information about the anti-allergic activities of Lamiaceae species. In addition, the botanical features, medicinal uses, chemical constituents and toxicological studies of Lamiaceae species were also documented. The method employed for data collection in this review was mainly the exploration of the PubMed, Ovid and Scopus databases. Additional research studies were obtained from the reference lists of retrieved articles. This comprehensive summarization serves as a useful resource for a better understanding of Lamiaceae species. The anti-allergic mechanisms related to Lamiaceae species are also reviewed extensively which aids in future exploration of the anti-allergic potential of Lamiaceae species.

## Introduction

Allergy is one of the manifestations of an abnormal regulation of the immune system. It can present as a mild to severe disorder, such as allergic rhinitis, food allergies, asthma, conjunctivitis, angioedema, urticaria, eczema, insect allergies and life-threatening anaphylaxis (Galli et al., 2008). Nowadays, allergy has become a global health concern. The cases of allergic disorders are increasingly rising and have reached an alarming rate. This statement is supported by some of the statistical figures provided by Pawankar (2014). According to Pawankar (2014), there are roughly 300 million individuals experiencing asthma and approximately 200 to 250 million individuals experiencing food allergies around the world. Additionally, 1/10 of the population experienced medication hypersensitivities and around 400 million people suffered from allergic rhinitis. Allergy can be very irritating to an extent that can greatly affect the quality of life, lead to an economic burden and can even jeopardize one’s life (Pawankar, 2014). Seeing the staggering pattern of morbidity and mortality caused by allergic disorders, this health issue must not be neglected and must be taken seriously with the active involvement of patients and healthcare professionals.

Currently, there are many treatment options for allergic disorders. Some of the widely used therapeutics are anti-histamine drugs, corticosteroids, leukotriene inhibitors and mast cell stabilizers to treat and control allergic conditions (Cota et al., 2012). All these medications are found to be efficactive in alleviating allergic symptoms. However, the drugs do not actually cure the allergy conditions. Instead, long term consumption of such drugs has been associated with undesirable side effects and sometimes may worsen the conditions (Han et al., 2017). Some examples of common side effects encountered by anti-histamine agents users are dry mouth, drowsiness, gastrointestinal disturbances, headache, agitation, confusion, etc. (Simon and Simons, 2008). As for corticosteroids, they work effectively in relieving allergic disorders, like allergic asthma, eczema, allergic rhinitis, etc. However, they often brings about undesirable side effects to patients in a long-term therapy. For instance, patients who are using inhaled corticosteroids for asthma control are likely to encounter undesirable effects like oral candidiasis (Fukushima et al., 2003) and adrenal suppression (Robert Webb, 1981). Patients with eczema who usually use topical steroidal treatment can develop Cushing’s syndrome (Tiwari et al., 2013) and skin thinning (Atherton, 2003) as well as easy bruising (Coondoo et al., 2014). Due to the limitations of modern medicines, there is an increasing interest in using complementary and alternative medicine, particularly herbal medicine for allergy conditions management (Engler et al., 2009).

Undeniably, medicinal plants have been widely utilized as healing modalities for both preventive and curative purposes. They play an extremely crucial role in human health. In recent years, there has been a growing trend in the world population with as many as 80% of people globally relying on the use of herbal medicinal products and supplements for their primary healthcare needs (Schuster, 2001; Ekor, 2013). This increasing demand and interest in the use of herbal medicinal products has encouraged new drug discoveries and developments (Ekor, 2013). In fact, many active ingredients of new drugs are derived from medicinal plants proven to be remarkably important in aiding drug discovery and development (Katiyar et al., 2012). Hence, studies need to be actively conducted on plants in order to identify possible candidates as safer and effective anti-allergic agents in future.

The Lamiaceae family is one of the biggest plant families among flowering plants, consisting of 236 genera with a coverage of more than 7,000 species (Khoury et al., 2016). It is also an important herbal family which comprises a wide array of plants with biological and medical applications (Uritu et al., 2018). Lamiaceae species often have four-angled or quadrangular stems with the presence of glandular hairs (Harley et al., 2004). Their roots are usually made of branched tap root. Their flowers are typically hypogynous and bilaterally symmetric with five united petals and sepals (Kokkini et al., 2003; Ramasubramania Raja, 2012; Carović-Stanko et al., 2016). The leaves are simple and arranged oppositely, each pair at a right angle to the previous one or whorled (Harley et al., 2004). Fruits are made of four dry one-seeded nutlets (Kokkini et al., 2003). Seeds are non-endospermic (Ramasubramania Raja, 2012). The environment adaptation of Lamiaceae is highly varied. The species predominantly distribute in the summer rainfall areas but also occur in winter rainfall areas. The species usually can be found in habitats which are dry, rocky, woodland or grassland, along forest margins and in fynbos (Will and Claßen-Bockhoff, 2014). The diversity of Lamiaceae species is mainly concentrated in Mediterranean regions and a small portion of them inhabit Australia, Southwest Asia and South America (Kokkini et al., 2003).

Khoury et al. (2016) reported that the high content of volatile compounds has contributed to many medicinal properties in Lamiaceae species. Historically, Lamiaceae plants have been reported to be effective in alleviating a range of conditions like exhaustion, weakness, depression, memory enhancement, circulation improvement, strengthening of fragile blood vessels, skin allergies and asthma (Wang et al., 2004; Naghibi et al., 2010; Ramasubramania Raja, 2012). In the Eastern Himalayan region of India, several Lamiaceae species have been utilized traditionally to treat certain conditions. For instance, the leaves of *Clerodendrum serratum* have been used as a traditional remedy for eye disorders. Moreover, the leaves of *Elsholzia blanda* is used to relieve itching conditions. The seed of *Perilla frutescens* is also claimed to be effective against fever and headache (Kala, 2005). Meanwhile, in China, the Chinese tea brewed using the leaves of *Salvia officinalis* is used as a traditional remedy to treat tonsillitis and hypertension (Li et al., 2013). Another Lamiaceae species, *Scutellaria baicalensis* has been extensively used as traditional Chinese medicine (TCM) for thousands of years. It is known as Huang Qin in Chinese. The decoction prepared from dried roots is used as a traditional remedy for diarrhea, dysentery, hypertension, hemorrhaging, insomnia, inflammation and respiratory infections (Zhao et al., 2016). In Mediterranean regions, like Lebanon, *Mentha spicata* is formulated into infusions to ease digestive disorders, arthritis, gastritis. The infusion is also used as an antiemetic and antimicrobial agent (Khoury et al., 2016). The medicinal uses of commonly used Lamiaceae species are summarized in **Table 1**.

**Table 1:** Medicinal uses of commonly used Lamiaceae species.

Plant name	Country/region	Local name/common name	Medicinal use	Plant part used	Mode of preparation	References
Clerodendrum petasites (Lour.) S.Moore	Thailand	Thao yaai mom	Asthma	Aerial part	The aerial part is prepared as tea or alcoholic extract.	(Hazekamp et al., 2001)
Clerodendrum serratum (Linn.) Moon	Arunachal Pradesh, India	No information	Eye disorders	Leaves	No information.	(Kala, 2005)
Elsholtzia blanda (Benth.) Benth.	Arunachal Pradesh, India	No information	Itching conditions	Leaves	No information.	(Kala, 2005)
Epimeredi indica (L.) Rothm	China	Guang Fan Feng	Rheumatoid arthritis, bones and muscles ache, skin ulcer, hemorrhoids, eczema	Whole plant	The whole plant is used to prepare as medicinal bath.	(Li et al., 2006)
Mentha arvensis Linn.	Western Himalayas	Pudina	Stomach problems, allergy, liver and spleen disease, asthma, indigestion, rheumatic pains, arthritis	Leaves	Leaves are made as salad and formulated into infusion respectively.	(Khan and Khatoon, 2007)
	Korea	Bak-ha	Analgesic, local vasodilator, skin irritant, antispasmodic agent, acute mastitis, allergic dermatitis and skin itching	Aerial part	Sometimes combine with other herbs as traditional remedy.	(Shin, 2003)
Mentha longifolia (L.) L.	West Bengal, India	Junglipudina	Menstrual disorders, pulmonary infection, congestion, asthma, urinary tract infections, indigestion, back pain, headache and to fasten wound healing process	Leaves	The leaves are formulated into extract.	(Sinhababu and Arpita, 2013)
	India	No information	Carminative, stimulant, antiseptic and febrifuge	Leaves and flower tops	No information.	(Sinhababu and Arpita, 2013)
Mentha spicata Linn.	Thessaloniki, Greece	No information	Common cold and cough	Aerial part	No information.	(Karousou et al., 2007)
	Lebanon	No information	Digestive disorders, arthritis, gastritis, antiemetic and antimicrobial agents	No information	Formulated as infusions.	(Khoury et al., 2016)
Perilla frutescens (Linn.) Britton	India	No information	Arthritis	Seed oil	The oil is extracted from the plant seed and massaged onto the arthritis part.	(Singh, 1997)
	Arunachal Pradesh, India	No information	Fever and headache	Seed	No information.	(Kala, 2005)
Prunella vulgaris Linn.	Iberian Peninsula	No information	External antiseptic	Aerial part	No information.	(Rigat et al., 2015)
Salvia miltiorrhiza Bunge	China	Danshen	Promoting cardiovascular health by improving blood circulation to remove blood stasis, clearing heart heat to relieve restlessness and cooling blood to remove carbuncle	Root	The root is air-dried and made into decoctions and pills. Nowadays, the root is widely formulated into various preparations, such as tablets, capsules, granules, injections, oral liquids, sprays and tea.	(Su et al., 2015)
Salvia officinalis Linn.	China	No information	Tonsillitis and hypertension	Leaves	The leaves are brewed as tea.	(Li et al., 2013)
Salvia plebeia R. Brown	Korea	Baem-Cha-Zu-Ki	Skin inflammatory disease and asthma	No information	No information	(Choi et al., 2014; Shin and Kim, 2002)
Scutellaria baicalensis Georgi	China	Huang Qin	Diarrhea, dysentery, hypertension, hemorrhaging, insomnia, inflammation and respiratory infections	Root	The dried root is used to prepare decoctions.	(Zhao et al., 2016)
Thymus serpyllum Linn.	Uttar Pradesh, India	No information	Headache, dysentery and vomiting	No information	Prepared as decoction.	(Singh, 1997)
Thymus vulgaris Linn.	Indonesia	No information	Asthma and other respiratory disorders	Leaves	No information	(Ikawati et al., 2001)
Vitex negundo Linn.	Uttar Pradesh, India	No information	Pain, swelling and eye inflammation	Leaves	The leaves are prepared as paste and applied onto the sprains to relieve pain. The leaf juice is used as drops to reduce eye inflammation.	(Singh, 1997)
Vitex trifolia Linn.	Indonesia	No information	Asthma and other respiratory disorders	Leaves	No information	(Ikawati et al., 2001; Alam et al., 2002)

This review is particularly focused on the summarization of the anti-allergic activities of the Lamiaceae family linked to the phytochemistry and ethnopharmacology reported in research studies. In addition to anti-allergic activities, toxicological investigations of Lamiaceae species are also highlighted in this review.

## Anti-Allergic Activity

**Figure 1:**
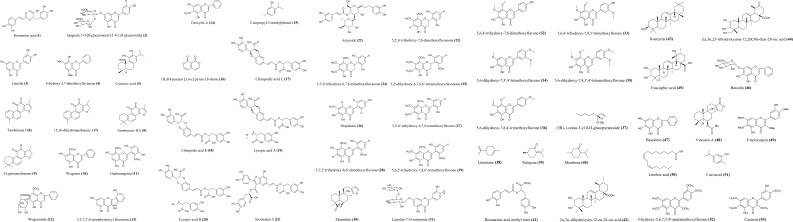
Chemical structures of phytochemicals isolated from Lamiaceae species with anti-allergic activity.

The abundance of species within the Lamiaceae family has led to a variety of medicinal uses, making the family pharmacologically important. The diversity is believed to be due to the wide variety of biologically active constituents in this plant family. Each species comprises a mixture of phytochemicals which attributes to the bioactivity of the plant (Carović-Stanko et al., 2016). Phytochemical investigations of the Lamiaceae family have demonstrated the presence of various bioactive compounds such as flavonoids (da Silva et al., 2015; Mamadalieva et al., 2017; Aghakhani and Kharazian, 2018), alkaloids (Malik et al., 2003; Asghari et al., 2017), phenolics (Berdowska et al., 2013; Zielińska and Matkowski, 2014; Skendi et al., 2017), lignans (Hong et al., 2009; Brandão et al., 2017), terpenoids (Ye et al., 2018), saponins (Ramasubramania Raja, 2012; Shah et al., 2014), etc. All these chemical constituents contribute to multidirectional pharmacological activities. Some of the remarkable bioactivities reported within this plant family are anti-allergic (Malik et al., 2003; Makino et al., 2003; Kim et al., 2009), anti-inflammatory (Borges et al., 2018), antimicrobial (Khoury et al., 2016; Cocan et al., 2018), free radical scavenging (Khaled-Khodja et al., 2014; Politeo et al., 2018), antinociceptive (Hwang et al., 2018; Uritu et al., 2018), anti-cancer activities (Nguyen et al., 2018; Sajjadi et al., 2018), etc. Many pharmacological activities of the Lamiaceae family have been widely studied and investigated. However, this study is mainly focused on the potential biologically active candidates with promising anti-allergic activity from Lamiaceae species in order to provide a direction in the discovery of potential novel, safe and efficacious natural anti-allergic agents in future. In the past, numerous *in vitro*, *in vivo* and *ex vivo* studies have been conducted and evaluated on the plant parts of Lamiaceae species to investigate the anti-allergic potential of Lamiaceae plants. **Figure 1** and **Table 2** show a summarization of the remarkable anti-allergic activities of the Lamiaceae family. The mechanisms of anti-allergic activities of Lamiaceae species are extensively discussed in this review.

**Table 2:** Mechanism of action of extracts and isolates of Lamiaceae species with anti-allergic activity.

Plant name	Plant part used	Isolated compound/ extract used	Chemical class	Assay type	Mechanism of action/conclusion	References
Clerodendron phlomidis Linn.	Leaves	Aqueous extract	–	In vivo	Concentration of 100 mg/kg potently reduced blood eosinophil count, mast cell degranulation and histamine release in sensitized mice.	(Vadnere et al., 2007)
				Ex vivo	Potent antagonizing effect of histamine-induced goat tracheal contraction at doses of 4 mg/ml and 10 mg/ml.	(Vadnere et al., 2007)
Clerodendrum serratum (Linn.) Moon	Root and stem	Aqueous extract	–	In vivo	Root extract resulted in no significant increase of leucocyte and eosinophil count at 260 mg/kg in milk-induced leucocytosis mice and prolonged PCD at 156 mg/kg in egg albumin-sensitized guinea pigs.	(Bhangare et al., 2012)
Clerodendron trichotomum Thunb.	Leaves	Acteoside **(22)**	Phenylpropanoid glycoside	In vivo	At dose of 50 mg/kg significantly inhibited eosinophil infiltration, decreased histamine content and phospholipase A_2_ activity in BALF while at 25 mg/kg, recruitment of leukocytes was suppressed and inhibited sRaw in both IAR and LAR in sensitized guinea pigs model.	(Lee et al., 2011b)
Clinopodium gracile (Benth.) Matsum var. multicaule	Whole plant	Aqueous extract	–	In vivo	Compound 48/80-induced mice were observed with concentration-dependently reduced anaphylactic death with intraperitoneally administration at concentrations ranging from 1-100 mg/kg and the same reduction manner was seen in IgE-mediated PCA reaction.	(Park et al., 2010)
				In vitro	Dose-dependent inhibition of histamine release inhibition from RPMC and HMC-1 cells respectively across 1-100 µg/ml. Allergic inflammation reduced with the attenuation of intracellular calcium, NF-κB, gene expression and secretion of TNF-α and IL-6 stimulated by PMACI in HMC-1 cells.	(Park et al., 2010)
Dracocephalum argunense Fisch.	Whole plant	Aqueous extract	–	In vivo	Significant inhibition of systemic anaphylaxis with intraperitoneal administration of aqueous extract in mice at concentration range of 0.01–1 g/kg. Serum histamine and PCA inhibition were reduced in a dose-dependent manner.	(Kim et al., 2006; Kim and Shin, 2006)
				In vitro	Decreased intracellular calcium and histamine release from RPMC in dose dependent manner with concentrations of 0.001–1 mg/ml. TNF-α and IL-6 gene expression in HMC-1 cells were inhibited across doses ranging from 0.01–1 mg/ml with the involvement of NF-κB attenuation.	(Kim et al., 2006; Kim and Shin, 2006)
Elsholtzia ciliate (Thunb.) Hyland	Whole plant	Aqueous extract	–	In vivo	Serum histamine, systemic anaphylaxis and PCA reaction observed with dose-related inhibition at concentrations of 10–1,000 mg/kg.	(Kim et al., 2011)
				In vitro	Significantly inhibited histamine release at 10 and 100 µg/ml of aqueous extract and recorded with reduction of PMACI-stimulated intracellular calcium at 100 µg/ml of aqueous extract pretreatment in HMC-1 cells. Gene expression and production of IL-6, IL-1β and TNF-α were suppressed through inhibition of NF-κB activation and p38 MAPK pathway with concentrations ranging from 1-100 µg/ml.	(Kim et al., 2011)
Isodon japonicas Hara	Whole plant	Aqueous extract	–	In vivo	Challenged-mice protected from systemic allergic death and PCA with intraperitoneal administration of extract at concentration of 0.1 g/kg.	(Kim et al., 2004; Shin et al., 2004)
				In vitro	Dose-dependently decreased histamine release from RPMC stimulated by compound 48/80 or anti-DNP IgE at doses ranging from 0.001-1 mg/ml and reduced gene expression and production of TNF-α and IL-6 in PMACI-stimulated HMC-1 cells.	(Kim et al., 2004; Shin et al., 2004)
Lagochilus leiacanthus Fisch. & C.A.Mey.	Whole plant	5,2’,6’-trihydroxy-7,8-dimethoxyflavanone **(23)**, 5,2’,6’-trihydroxy-6,7,8-trimethoxyflavanone **(24)**, 5,2’-dihydroxy-6,7,8,6’-tetramethoxyflavanone **(25)**, Oroxylin-A **(14)**, Hispidulin **(26)**, 5,2’,6’-trihydroxy- 6,7,8-trimethoxyflavone **(27)**, 5,7,2’-trihydroxy-8,6’- dimethoxyflavone **(28)**, 5,6,2’-trihydroxy-7,8,6’-trimethoxyflavone **(29)**	Flavonoid	In vitro	Significantly inhibited the release of β-hexosaminidase from RBL-2H3 cells with IC_50_ values ranging from 13.5-48.9 µM.	(Furukawa et al., 2011)
Lycopus lucidus Turcz.	Aerial part	Rosmarinic acid **(1)**, clinopodic acid C **(17)**, lycopic acid A **(19)**, clinopodic acid E **(18)**, lycopic acid B **(20)**, scizotenuin A **(21)**	Phenylpropanoid	In vitro	Acted as hyaluronidase inhibitor with IC_50_ of 309, 80.1, 134, 82.8, 141 and 241 µM respectively.	(Murata et al., 2010)
	Whole plant	Aqueous extract	–	In vivo	Systemic anaphylactic death and PCA reaction reduced dose-dependently across concentrations of 0.005–0.1 g/kg in sensitized mice.	(Shin et al., 2005)
				In vitro	Histamine release potently reduced in compound 48/80 or anti-DNP IgE-stimulated RPMC corresponded to decreased intracellular calcium at dose range of 0.01–1 mg/ml. Attenuation of NF-κB caused a reduction in downstream cytokines, such as TNF-α and IL-6 expression.	(Shin et al., 2005)
Marrubium vulgare Linn.	Aerial part	Marrubiin **(30)**	Furane labdane diterpene	In vitro	Maximal inhibition (67.6 ± 4%) of OVA-induced allergic oedema was achieved at dose of 100 mg/kg in actively sensitized mice.	(Stulzer et al., 2006)
Melissa officinalis Linn.	Leaves	Rosmarinic acid **(1)**	Phenylpropanoid	In vitro	Exhibited potent suppressive effect on hyaluronidase with 1.0 ± 0.3% of enzyme activity.	(Ippoushi et al., 2000)
Mentha arvensis Linn.	Leaves, roots and stem	Ethanolic and aqueous extract	–	In vivo	100 µg/ml ethanolic leaf and root extract showed potent histamine inhibition at 57% and 53% respectively in mice.	(Malik et al., 2003)
	Whole plant	Aqueous extract	–	In vivo	Anal administration of 0.05 g/kg extract protected mice from anaphylactic death. PCA reaction reduced dose-dependently with intraperitoneal, oral and intravenous administration of extract.	(Shin, 2003)
				In vitro	Significant reduction in histamine release at 0.1 and 1 mg/ml of extract in compound 48/80-induced and anti-DNP IgE-mediated model in RPMC. TNF-α production reduced significantly at concentration of 0.1 mg/ml in RPMC.	(Shin, 2003)
	–	Essential oil	–	In vivo	Dose-dependently reduced histamine-induced bronchoconstriction in guinea pigs at 200 and 400 µl/kg and significantly reduced eosinophil count, serum IgE level and BALF eosinophils in OVA-sensitized mice at 200 µl/kg.	(Sharma et al., 2018)
Mentha haplocalyx Briq.	Aerial part	Ethanolic extract	–	In vivo	Observed with inhibition of eosinophil infiltration, T_H_2 cytokines (IL-4, IL-5) expression and production in OVA-sensitized mice.	(Lee et al., 2011a)
Mentha piperita Linn.	Leaves	50% ethanolic eluate	–	In vivo	Decreased nasal responses in antigen-induced rats at 300 and 1,000 mg/kg.	(Inoue et al., 2001)
				In vitro	Recorded with potent inhibition of histamine release from RPMC with IC_50_ of 2.55 (1.42–3.94) µg/ml.	(Inoue et al., 2001)
	Leaves	Luteolin-7-O-rutinoside **(31)**	Flavonoid glycoside	In vivo	Reduced frequency of sneezing at 100 and 300 mg/kg while nasal rubbing was seen in antigen-induced rats at 100 mg/kg or more.	(Inoue et al., 2002)
				In vitro	Suppression of histamine release from compound 48/80-induced RPMC with IC_50_ value of 21.9 µM.	(Inoue et al., 2002)
Mentha piperita var. citrata (Ehrh.) Briq.	Leaves	5,6,4’-trihydroxy-7,8-dimethoxyflavone **(32)**, 5,6,4’-trihydroxy-7,8,3’- trimethoxyflavone **(33)**, 5,6-dihydroxy-7,3’,4’- trimethoxyflavone **(34)**, 5,6-dihydroxy-7,8, 3’, 4’- tetramethoxyflavone **(35)**, 5,6-dihydroxy-7,8, 4’-trimethoxyflavone **(36)**	Flavonoid	In vitro	Significantly reduction of β-hexosaminidase release from RBL-2H3 at IC_50_ range of 2.4–6.7 µM.	(Sato and Tamura, 2015)
Mentha spicata L. var. crispa Benth.	Leaves	5,6-dihydroxy-7,8,3’,4’-tetramethoxyflavone **(35)**, 5,6,4’-trihydroxy-7,8,3’-trimethoxyflavone **(33)**, (3R)-1-octan-3-yl β-D-glucopyranoside **(37)**	Flavonoid and aliphatic glycoside	In vivo	Demonstrated β-hexosaminidase release suppression from rat basophils at 56, 6.4 and 560 µM respectively.	(Yamamura et al., 1998)
Minthostachys verticillata (Griseb.) Epling	Leaves and stems	Essential oil	–	In vitro	β-hexosaminidase release from human basophils was diminished by 32.15% to 39.72% as comparable to dexamethasone and theophylline.	(Cariddi et al., 2007)
	Leaves and stems	Limonene **(38)**, Pulegone **(39)**, Menthone **(40)**	Monoterpene	In vivo	**(38)** maximally suppressed PCA reaction in challenged-mice at 250 mg/kg.	(Cariddi et al., 2011)
				In vitro	The combination of the three constituents in essential oil significantly suppressed the production of IL-13 from human PBMC. Potent inhibitory effect on β-hexosaminidase release from human basophils was observed with a concentration range of 10–40 µg/ml.	(Cariddi et al., 2011)
Mosla chinensis Max.	Whole plant	Aqueous extract	–	In vivo	Observed with concentration dependent suppression of systemic anaphylaxis and PCA reaction with doses of ranging from 10–1,000 mg/kg in sensitized mice.	(Kim et al., 2012)
				In vitro	Decreased intracellular calcium caused a dose-dependent reduction of histamine release from RPMC and reduced NF-κB activation resulted in decreased downstream TNF-α, IL-6, IL-8 expression in PMACI-stimulated HMC-1 cells.	(Kim et al., 2012)
Mosla dianthera Maxim.	Whole plant	Aqueous extract	–	In vivo	Challenged-mice recorded with zero mortality due to systemic shock with pretreatment of 1,000 mg/kg and reduced PCA reaction in a dose-dependent manner across the range of 1–1,000 mg/kg.	(Lee et al., 2006)
				In vitro	Attenuation of histamine release from RPMC at doses ranging from 0.001–1 mg/ml and decreased intracellular calcium, NF-κB activation, gene expression and secretion of TNF-α, IL-6, IL-8 in PMACI-stimulated HMC-1 cells.	(Lee et al., 2006)
Nepeta bracteata Benth.	Whole plant	Crude aqueous extract	–	In vivo	Medium dose (not specified dose) exhibited the most potent reduction in the number of T_H_17 cells, increased number of Treg cells in OVA-sensitized mice and decreased eosinophil infiltration in BALF.	(Wang et al., 2016)
Ocimum basilicum Linn.	Leaves	Acetone and hydro-methanol extract	–	In vitro	Achieved histamine release suppressive effect of 35.35% and 50.76% respectively at 100 µg/ml.	(Kaur et al., 2018)
Ocimum gratissimum Linn.	Leaves	Methanolic extract	–	In vivo	Significantly suppressed number of eosinophils, decreased IL-4 level, reduced level of eosinophil peroxidase in BALF and lungs and decreased airway mucus hypersecretion at 100 mg/kg.	(Costa et al., 2012)
Ocimum sanctum Linn.	Leaves and seeds	Dried and fresh leaves ethanolic extract, volatile oil from fresh leaves and fixed oil from seeds	–	In vivo	Guinea pigs were protected from both histamine-induced and acetylcholine-induced preconvulsive dyspnoea with pretreatment of fresh leaves ethanolic extract, volatile oil and fixed oil.	(Singh and Agrawal, 1991)
	Leaves	Ethanolic extract	–	In vivo	Exhibited significant mast cell stabilizing potential, inhibition of IgE and delayed onset of histamine-induced bronchospasm with 64.25 ± 9.51%, 25.80 ± 4.85 ng/ml and 440 s respectively.	(Sridevi et al., 2009)
	Leaves	Ethanolic extract and isolated flavonoidal fraction	–	In vivo	Sensitized rats showed with significant mast cell stabilization of 67.24 ± 2.94% with extract administration and 60.48 ± 2.72% with fraction administration.	(Choudhary, 2010)
Perilla frutescens Britton	Whole plant	Aqueous extract	–	In vivo	Exhibited dose-dependent inhibition of compound 48/80-induced plasma histamine release at concentration range of 0.01–1 g/kg in sensitized rats. Marked suppression of PCA reaction at doses of 0.1 and 1 g/kg.	(Shin et al., 2000)
				In vitro	At concentration range of 0.001–1 mg/ml, histamine release and TNF-α production were decreased dose-dependently in stimulated RPMC. cAMP level in RPMC significantly increased at 1 mg/ml.	(Shin et al., 2000)
	Whole plant	Rosmarinic acid **(1)**, Apigenin 7-O-[β-glucuronosyl(2→1) β-glucuronide] **(2)**	Flavonoid and flavonoid glycoside	In vivo	**(1)** and **(2)** suppressed PCA reaction in sensitized mice with percentage inhibition of 41% and 32% respectively.	(Makino et al., 2001)
	Leaves	Luteolin **(3)**	Flavonoid	In vivo	Sensitized mice showed with decreased oxazolone-induced ear odema with oral administration of 1 mg of **(3)**. Dose-dependent inhibition of TNF-α production occurred at dose range of 1–1,000 µg.	(Ueda et al., 2002)
	Leaves	Rosmarinic acid **(1)**	Phenylpropanoid	In vivo	Equivalent PCA reaction suppression was achieved by 19 mg/kg of **(1)** as compared to 150 mg/kg of tranilast, the positive control.	(Makino et al., 2003)
	Leaves	Rosmarinic acid **(1)**	Phenylpropanoid	In vivo	Oral administration of **(1)** by Der f-sensitized mice caused reduction in allergen-specific immunoglobulin, eosinophil infiltration, eosinophil counts in BALF, eotaxin, IL-4 and IL-5 expression at concentration of 1.5 mg/day.	(Sanbongi et al., 2004)
	Leaves	Ethanol extract	–	In vivo	T_H_2 cytokines (IL-5 and IL-13), serum IgE level, eosinophil infiltration, histamine and eotaxin in BALF were suppressed in OVA-sensitized BALB/c mice.	(Chen et al., 2015)
	Leaves	Luteolin **(3)**	Flavonoid	In vivo	Compound 48/80- or serotonin-induced scratching behaviour and vascular permeability were reduced dose-dependently at 5, 10 and 20 mg/kg in sensitized mice.	(Jeon et al., 2014)
				In vitro	Dose-dependent reduction of compound 48/80-induced histamine release from RPMC marked at 5, 10 and 20 µM. Diminished production of TNF-α (31.9%-76.8%) and IL-1β (27.3%-81.2%) in PMACI-stimulated HMC-1 cells at a range of 5–20 µM of **(8)**.	(Jeon et al., 2014)
	Leaves	Aqueous fraction	–	In vivo	DNFB-sensitized mice experienced 35% reduction of ear swelling symptom at dose of 100 µg/ml.	(Heo et al., 2011)
				In vitro	At 100 µg/ml, eosinophil counts reduced by 73.7% accompanied with decreased expression of MMP-9 and IL-31 in mice ear tissues. T-bet protein expression was augmented and resulted in T_H_1/T_H_2 balance.	(Heo et al., 2011)
	Leaves	Methanolic extract	–	In vitro	Dose-dependent reduction of IL-4, IL-5, IL-13 and GM-CSF production in DP2-stimulated BEAS-2B cells at concentration range of 5-50 µg/ml with involvement of decreased phosphorylation of JNK and p38.	(Liu et al., 2013)
	Leaves	Rosmarinic acid methyl ester **(41)**	Phenolic compound	In vitro	Possessed potent inhibitory activity on β-hexosaminidase from RBL-2H3 cells with IC_50_ of 9.9 ± 0.8 µg/ml.	(Zhu et al., 2014)
	Leaves	8-hydroxy-5,7-dimethoxyflavanone **(4)**	Flavanone	In vivo	Oral administration of **(4)** inhibited PCA reaction at dose of 5 mg and allergic nasal response at dose of 1.5 mg.	(Kamei et al., 2017)
				In vitro	Significant suppressive effect on histamine release from RBL-2H3 cells was observed with IC_50_ of 68.5 µM.	(Kamei et al., 2017)
	Leaves	Crude extract	–	In vivo	Decreased serum IgE level was observed in the blood of Der f-challenged mice.	(Komatsu et al., 2016)
				In vitro	Reduced CD4^+^/CD8^+^ ratio in splenic T lymphocytes with percentage of 1.50 ± 0.07%.	(Komatsu et al., 2016)
Phlomis umbrosa Turcz.	Roots	Aqueous extract	–	In vivo	At dose of 1 g/kg, plasma histamine release only recorded with 0.023 ± 0.002 µg/ml in compound 48/80- sensitized mice. Anal, oral and intraperitoneal administration of 0.01–1 g/kg extract resulted in dose-dependent reduction in PCA reaction.	(Shin et al., 2008; Shin and Lee, 2003)
				In vitro	Significant inhibition of histamine release from RPMC activated by compound 48/80 or anti-DNP IgE recorded at doses of 0.1 mg/ml and 1 mg/ml. Extract attenuated the secretion of IL-1β, IL-6 and TNF-α in PMACI-stimulated HMC-1 cells.	(Shin et al., 2008; Shin and Lee, 2003)
Pogostemon cablin (Blanco) Benth	–	Aqueous extract	–	In vivo	Intraperitoneal administration of extract caused a dose-related suppression of systemic anaphylaxis induced by compound 48/80 in sensitized mice across doses of 10–1,000 mg/kg. At doses ranging from 1–1,000 mg/kg, PCA reaction induced by DNP-HSA was reduced in a concentration-dependent manner in sensitized rats.	(Yoon et al., 2016)
				In vitro	Suppressed the release of histamine and β-hexosaminidase from RPMC across concentrations of 1–1,000 µg/ml. Expression and secretion of TNF-α, IL-6 and IL-8 was inhibited in HMC-1 cells at doses of 1–100 µg/ml due to the attenuation of NF-κB activation.	(Yoon et al., 2016)
	–	Patchouli oil	–	In vivo	Significant reduced PCA reaction in ovalbumin-challenged rats and suppressed delayed-type hypersensitivity at doses of 20, 40 and 80 mg/kg of Patchouli oil.	(He et al., 2013)
				Ex vivo	Decreased contraction responses in guinea pig ileum at concentrations of 0.01, 0.02 and 0.04 mg/ml of Patchouli oil.	(He et al., 2013)
Prunella vulgaris var. lilacina Nakai	Whole plant	2α,3α-dihydroxyurs-12-en-28-oic acid **(42)**	Triterpenoid	In vitro	Significant suppressive effect on β-hexosaminidase from RBL-2H3 with IC_50_ value of 57 µM.	(Ryu et al., 2000)
	Whole plant	Aqueous extract	–	In vivo	At doses of 0.5 and 1 g/kg, sensitized rats completely protected from anaphylatic death. Oral administration of extract with doses ranging from 0.001–1 g/kg dose-dependently reduced PCA reaction.	(Kim et al., 2007; Shin et al., 2001)
				In vitro	Inhibition of intracellular calcium level caused downstream decreased release of histamine from RPMC in a concentration dependent manner with concentration range of 0.001–1 mg/ml. RPMC also showed with significant reduction of TNF-α production with the pretreatment of 0.01 mg/ml and 0.1 mg/ml.	(Kim et al., 2007; Shin et al., 2001)
	Spike	β-amyrin **(43)**, 2α,3α,23-trihydroxyursa-12,20(30)-dien-28-oic acid **(44)**, Euscaphic acid **(45)**	Triterpenoid	In vitro	Observed with inhibition of histamine release from HMC-1 cells with 46.7%, 57.9% and 54.2% respectively.	(Choia et al., 2016)
Rosmarinus officinalis Linn.	Leaves	Carnosic acid **(5)**	Polyphenol	In vivo	PCA reaction was significantly suppressed at 100 mg/kg in sensitized mice with percentage inhibition of 67.1%.	(Mizushina et al., 2014)
				In vitro	**(5)** inhibited β-hexosaminidase release from PMACI A23187-stimulated RBL-2H3 cells at 10 µM.	(Mizushina et al., 2014)
Salvia miltiorrhiza Bunge	Roots	15,16-dihydrotanshinone-I **(7)**, Cryptotanshinone **(9)**	Diterpene	In vitro	**(7)** and **(9)** significantly suppressed the release of β-hexosaminidase from RBL-2H3 cells with IC_50_ values of 16 ± 2.4 µM and 36 ± 3.7 µM respectively.	(Choi and Kim, 2004; Ryu et al., 1999)
	Leaves	Ethanolic extract	–	In vivo	Oral administration of 25–100 mg/kg extract dose-dependently inhibited PCA reaction in anti-DNP IgE-stimulated rats.	(Yang et al., 2008)
				In vitro	Dose-related inhibition of COX-1 and COX-2-dependent prostaglandin D_2_ production observed with IC_50_ values of 3.96 and 21.54 µg/ml respectively in BMMC. Suppression of leukotriene C_4_ generation and β-hexosaminidase release was seen in BMMC with IC value of 2.6 and 22.4 µg/ml.	(Yang et al., 2008)
	Rhizome	Tanshinone I **(6)**, 15,16-dihydrotanshinone-I **(7)**, Tanshinone IIA **(8)**, Cryptotanshinone **(9)**	Diterpene	In vivo	**(6)**, **(7)**,** (8)** and **(9)** significantly suppressed PCA reaction at dose of 50 mg/kg with percentage inhibition of 59%, 49%, 35% and 32% respectively in sensitized mice.	(Trinh et al., 2010a)
				In vitro	Potent inhibition of IL-4 and TNF-α expression by **(6)**, **(7)** and **(8)** at dose of 50 µM in RBL-2H3 cells.	(Trinh et al., 2010a)
	Roots	15,16-dihydrotanshinone-I **(7)**	Diterpene	In vitro	**(7)** at 20 µM produced 90% suppression on degranulation and generation of prostaglandin D_2_ and leukotriene C_4_ in IgE/Ag-stimulated BMMC through inhibition of FcɛRI-mediated Syk-dependent signal pathway.	(Li et al., 2015)
Salvia plebeia R. Brown	Whole plant	Aqueous extract	–	In vivo	No anaphylactic death occurred in compound 48/80-induced rats with intraperitoneal administration of 0.5 and 1 g/kg extract. At doses ranging from 0.01–1 g/kg, intraperitoneal and oral administration of extract showed with dose-dependent inhibition of PCA reaction.	(Shin and Kim, 2002)
				In vitro	Concentration-dependent reduction histamine release from RPMC activated by compound 48/80 or anti-DNP IgE at a concentration range of 0.001–1 mg/ml. TNF-α production from RPMC was significantly inhibited at concentrations of 0.01–1 mg/ml whereas cAMP level in RPMC significantly elevated compared with that of basal cells.	(Shin and Kim, 2002)
	Whole plant	Ethanol extract	–	In vivo	Oral administration of 100 mg/kg extract significantly suppressed serum IgE level, serum histamine, eosinophil count, pro-inflammatory cytokines (IFN-γ and TNF-α) expression, T_H_1, T_H_2 and T_H_17 cytokines expression in Der f-sensitized mice.	(Choi et al., 2014)
Schizonepeta tenuifolia (Benth.) Briq.	Whole plant	Aqueous extract	–	In vivo	100% protection from systemic anaphylaxis was observed with doses of 0.5 and 1 g/kg in compound 48/80-challenged rats whereas a marked suppression in PCA reaction was seen in orally administered of 0.1 and 1 g/kg of anti-DNP IgE-sensitized rats.	(Shin et al., 1999)
				In vitro	Significant inhibition of compound 48/80 or IgE-mediated histamine release from RPMC was marked at concentration range of 0.01–1 mg/ml. A potent inhibition of TNF-α production observed at 1 mg/ml with a content of 0.889 ± 0.747 ng/ml.	(Shin et al., 1999)
	Whole plant	Extract with phosphate buffered saline/olive oil (P/O) in proportion of (9:1) mixture	–	In vivo	Skin thickening and hyperplasia of epidermis and dermis in DNCB-sensitized mice remarkably decreased by 38.15% and 42.37% respectively with treatment of 1% of extract in P/O (9:1) mixture.	(Choi et al., 2013)
				In vitro	DNCB-induced mice observed with reduced serum levels of IgE, TNF-α and IL-6, recorded with 46.26%, 41.97% and 70.42% inhibition respectively with the treatment of 1% of extract in P/O (9:1) mixture.	(Choi et al., 2013)
	–	Aqueous extract	–	In vitro	Pro-inflammatory cytokines (IL-6, IFN-γ and TNF-α) and pro-allergic T_H_2 cytokines (IL-4 and IL-13) in RBL-2H3 were decreased with the treatment of 100 µg/ml of aqueous extract. β-hexosaminidase release from RBL-2H3 cells reduced significantly at dose of 10 µg/ml.	(Lin et al., 2018)
Scutellaria baicalensis Georgi	Roots	Wogonin **(10)**, Ganhuangenin **(11)**, Wogonoside **(12)**, 3,5,7, 2’,6’-pentahydroxyflavanone **(13)**	Flavonoid	Ex vivo	**(10)**, **(11)** and **(12)** significantly inhibited the production of IgE from concanavalin A-stimulated rat spleen lymphocytes at concentrations of 10.0 and 100.0 µM. Histamine and leukotriene B_4_ release from rat PEC was markedly suppressed at dose of 100 µM for all flavonoids.	(Lim, 2003; Lim et al., 2003)
	Roots	Aqueous extract	–	In vivo	Oral administration of 50 mg/kg extract selectively inhibited the release of IL-5 in mice.	(Kim et al., 2010)
	Roots	Baicalin **(46)**	Flavone glycoside	In vitro	Histamine and leukotriene release from OVA-sensitized guinea pig lung mast cells were potently suppressed at doses of 10, 30 and 60 µg. The standardized extract of **(46)** exhibited a more potent outcome than pure **(46)** at 60 µg only.	(Kim et al., 2010)
	Whole plant	Ethanolic extract	–	In vivo	Exhibited 6.6% inhibition of PCA reaction in sensitized-rats at 280 mg/kg.	(Jung et al., 2012)
				In vitro	40% reduction of histamine content in compound 48/80-stimulated RPMC with dose of 10 µg/ml. Significant reduced production of TNF-α and IL-8 in PMACI-stimulated HMC-1 cells with inhibition of MAPK activation at concentration range of 1–100 µg/ml.	(Jung et al., 2012)
	Roots	Aqueous extract	–	In vivo	Topical application of 5% extract reduced DNFB-induced cutaneous reaction by 31% as compared to control group.	(Kim et al., 2013)
				In vitro	Significantly suppressed β-hexosaminidase release from RBL-2H3 at doses of 125, 250 and 500 ppm with percentage inhibition of 19%, 34% and 60% respectively.	(Kim et al., 2013)
	Whole plant	Crude ethanol extract	–	In vivo	OVA-sensitized mice were protected from food allergy anaphylactic death by 60% and observed with significantly suppression of OVA-specific IgE, IL-17, T_H_2 cytokines (IL-4, IL-5, IL-10, IL-13) and T_H_1 cytokines (IFN-γ and IL-12) with the treatment of 25 mg/kg of extract.	(Shin et al., 2014b)
	–	Wogonin **(10)**, Baicalin **(46)**, Baicalein **(47)**	Flavonoid	Ex vivo	**(10)** suppressed the production of T_H_2 cytokines (IL-4, IL-5, IL-10, IL-13) and IFN-γ without causing cytotoxicity in OVA-sensitized mice splenocytes at 50 µmol/ml as compared to **(46)** and **(47)**.	(Shin et al., 2014a)
				In vivo	Oral administration of 1 mg/kg of **(15)** potently decreased the production of OVA-specific IgE, IL-5, IL-10 and IL-13 in sensitized mice.	(Shin et al., 2014a)
	Roots	Aqueous extract	–	In vivo	Attenuation of DNCB-induced epidermal thickness, leukocytes infiltration, serum IgE, IL-4, IFN-γ and TNF-α production in BALB/c mice skin.	(Kim et al., 2016)
	Root	Ethanol extract, acetone extract and ethyl acetate extract	–	In vivo	The highest inhibitory activity against 4-AP-induced allergic skin pruritus, histamine-induced paw swelling, ear PCA reaction, anaphylaxis ear swelling and total serum IgE level was seen with 1.42 g/kg of ethanol extract in sensitized mice.	(Li et al., 2014)
	Rhizome	Baicalin **(46)**, Baicalein **(47)**, Oroxylin A **(14)**	Flavonoid	In vivo	**(46)** which was orally administered metabolized into **(47)** and **(14)**. Metabolite, **(14)** possessed a more potent anti-histamine activity, seen with significant reduced histamine-induced scratching behaviour and vascular permeability in sensitized mice at doses of 20 and 50 mg/kg.	(Trinh et al., 2010b)
				In vitro	Metabolite of **(46)**, **(14)** remarkably inhibited the contraction of guinea pig ileum with IC_50_ value of 0.28 mmol/L.	(Trinh et al., 2010b)
	–	Oroxylin A **(14)**	Flavonoid	In vivo	Significantly reduced eosinophils infiltration in BALF and airway hyperresponsiveness in OVA-sensitized mice. Potent attenuation of serum IgE level, T_H_2 cytokines (IL-4, IL-5 and IL-13) production and NF-κB activation with oral administration of 15, 30 and 60 mg/kg.	(Zhou et al., 2016)
	Roots	Linoleic acid **(50)**	Fatty acid	Ex vivo	Significantly suppressed the production of IL-4, IL-5, IL-10 and IL-13 but enhanced secretion of IFN-γ and IL-12, resulted in T_H_1/T_H_2 balance at 50 µg/L.	(Jung et al., 2017)
	–	Baicalein **(47)**	Flavonoid	In vivo	**(47)** induced CD4^+^ FOXP_3_^+^ T cell differentiation in ovalbumin-sensitized mice at concentrations of <10 µmol/L without causing cell death.	(Bae et al., 2016)
Stachys riederi var. japonica Miq.	Whole plant	Aqueous extract	–	In vivo	Dose-dependent inhibition was resulted in the occurrence of systemic anaphylaxis at concentration range of 0.005–1 g/kg and significant reduction of PCA reaction at concentrations of 0.1 and 1 g/kg.	(Shin, 2004)
				In vitro	At doses of 0.1 and 1 mg/ml, a significant decrease release of histamine from RPMC and diminished secretion of TNF-α and IL-6 from HMC-1 cells.	(Shin, 2004)
Teucrium japonicum Houttuyn	Whole plant	Aqueous extract	–	In vivo	Serum histamine release was significantly reduced at 100 and 1,000 mg/kg, with 500 mg/kg as the effective dose that completely protected compound 48/80-induced mice from systemic anaphylaxis. At doses ranging from 1–1,000 mg/kg, a dose-dependent inhibition of PCA reaction was resulted in anti-DNP IgE-challenged mice.	(Kim et al., 2009)
				In vitro	Significant reduction of compound 48/80-induced intracellular calcium and downstream histamine release from RPMC was observed at 1 mg/ml. Gene expression of TNF-α was diminished dose-dependently at 0.01–1 mg/ml with the involvement of NF-κB in PMACI-stimulated HMC-1 cells.	(Kim et al., 2009)
Thymus vulgaris Linn.	Leaves	2-isopropyl-5-methylphenol **(15)**	Monoterpenoid phenolic	In vivo	Dose-dependently inhibited recruitment of inflammatory cells, reduced airway hyperreponsiveness, suppressed level of OVA-specific IgE, T_H_2 cytokines in BALF at concentrations of 4, 8 and 16 mg/kg.	(Zhou et al., 2014)
	Leaves	n-hexane extract	–	In vivo	Portrayed intermediate inhibitory activity on histamine release with 46.22 ± 0.08%.	(Ikawati et al., 2001)
	–	2-isopropyl-5-methylphenol **(15)**, Carvacrol **(51)**	Monoterpenoid phenolic	In vivo	**(15)** and **(51)** reduced delayed-type hypersensitivity by 26% and 50% respectively in ovalbumin-sensitized mice.	(Gholijani and Amirghofran, 2016)
				Ex vivo	Both compounds led to reduction of IL-2, IFN-γ, IL-4, IL-17 level and T-bet expression but increased level of IL-10 and TGF-β in mice splenocytes cultures.	(Gholijani and Amirghofran, 2016)
Vitex negundo Linn.	Leaves	Aqueous subfraction of ethyl acetate fraction	–	In vitro	Mast cell stabilizing activity with 80.99 ± 0.7231% was observed in rat mesenteric mast cells at dose of 500 µg/ml.	(Patel and Deshpande, 2011)
	Leaves	5-hydroxy-3, 6, 7, 3’,4’-pentamethoxyflavone **(52)**	Flavonoid	In vivo	200 mg/kg extract demonstrated significant reduction of eosinophil count in BALF and serum bicarbonate level in egg albumin sensitized guinea pigs.	(Patel and Deshpande, 2013)
Vitex rotundifolia Linn.	Fruits	Aqueous extract	–	In vivo	Dose-dependent reduction of systemic anaphylaxis reaction in compound 48/80-induced rats across concentration range of 0.0001–1 g/kg. Significant suppression of PCA reaction marked at doses of 0.5 and 1 g/kg in sensitized rats.	(Shin et al., 2000)
				In vitro	Histamine release from RPMC was reduced in a dose-dependent manner at dose range of 0.001–1 mg/ml and marked inhibition of TNF-α production at 0.001 mg/ml.	(Shin et al., 2000)
	Fruits	1H,8H-pyrano[3,4-c]pyran-1,8-dione **(16)**	Pyran	In vivo	Serum IgE, eosinophil counts and IL-5 production in BALF were significantly suppressed by 43%, 82% and 34% respectively. IL-4 and IL-5 level significantly decreased in CD4^+^ T cells in T_H_2 skewed condition with treatment.	(Lee et al., 2009)
				In vitro	Eosinophil migration and eotaxin production were reduced by 48% and 70% respectively at 10 µg/ml in A549 cell media.	(Lee et al., 2009)
	Fruits	Casticin **(53)**	Flavonoid	In vitro	Up to 63% of eosinophil migration inhibition was observed in A549 cell media with pretreatment of 10 µg/ml. Eotaxin level was reduced from concentration range of 0.1–10 µg/ml in A549 cells.	(Koh et al., 2011)
	–	Aqueous extract	–	In vivo	Oral administration of 100 mg/kg extract caused 86% inhibition of eosinophilia, reduction of T_H_2 cytokines (IL-4, IL-5, IL-13) and TNF-α level in BALF and decreased serum IgE level in ovalbumin-sensitized mice.	(Bae et al., 2013)
Vitex trifolia Linn.	Leaves	n-hexane and ethanolic extract	–	In vitro	Both n-hexane and ethanolic extract highly suppressed histamine release by 80.13 ± 3.95 and 81.58 ± 0.24% respectively in RBL-2H3 cells.	(Ikawati et al., 2001)
	Leaves	Viteosin-A **(48)**, Vitexicarpin **(49)**	Flavonoid	Ex vivo	**(48)** and **(49)** respectively reduced histamine-induced tracheal contraction by 27.1% and 66.2% at 0.00013 M and percentage increased to 47.9% and 97.2% respectively when raised to 0.0004 M.	(Alam et al., 2002)
Zataria multiflora Boiss.	Whole plant	Hydro-ethanolic extract	–	In vitro	Observed with increment of Treg cells, T_H_1/T_H_2 ratio, IFN-γ/IL-4 ratio, IFN-γ and FOXP_3_ expression. Significant reduction of T_H_2 and T_H_17 cells and decreased expression of IL-4, IL-17 and TGF-β occurred in sensitized mice spleen cells.	(Kianmehr et al., 2017)
	Seeds	Hydro-ethanolic extract	–	In vivo	Increased IFN-γ and decreased IL-4 were resulted in ovalbumin-sensitized guinea pigs with the oral administration of extract.	(Boskabady et al., 2013)
				In vitro	Achieved T_H_1/T_H_2 balance with enhanced ratio of IFN-γ/IL-4 in PHA-stimulated hPBMC.	(Boskabady et al., 2013)

## Inhibition of Allergen-Specific IgE

Immunoglobulin E (IgE) is of central importance in the regulation of immune responses against parasitic infestations and most importantly, it is also recognized as a main mediator for immediate-type allergies or type I hypersensitivity reactions, such as allergic asthma, rhinitis, atopic diseases, anaphylaxis, etc. (Al-Mughales, 2016). It is a potent mast cell activator able to trigger mast cell degranulation and downstream responses with a minute amount (Actor, 2014). It exists in trace amounts in plasma but the amount can be substantially elevated in allergic reactions (Gould and Beavil, 1998; van der Burg et al., 2014). It possesses an additional constant region, CH_4_, which particularly restricts it to bind to high affinity IgE receptors on mast cells and basophils (Flaherty, 2012). Upon the first encounter with the antigen, the plasma cells start to produce IgE molecules. The secreted IgE molecules bond to the high affinity IgE receptors (FcεRI) on the mast cells and basophils surfaces via their Fc portion, forming IgE-FcɛRI complexes. In this form, the half-life of IgE can be prolonged to two to three weeks or sometimes can even be retained on the cell surface for months (Actor, 2014; van der Burg et al., 2014). Upon the re-exposure to the same antigen, the antigen will cross-link with the IgE-FcεRI complexes which then lead to mast cell degranulation (Actor, 2014). Inhibition of IgE production and IgE-mast cell cross-linking are particularly essential to prevent the progression to mast cell degranulation.

There are several studies demonstrating that Lamiaceae species have a suppressive effect on IgE levels and IgE-mast cell cross-linking. These findings could be useful to recognize potential treatment options for allergic disorders. From the study of Sharma et al. (2018), results revealed that the essential oil of *Mentha arvensis* significantly decreased (P < 0.001) the serum IgE level in OVA-sensitized mice at a concentration of 200 µl/kg. The study successfully identified three compounds in the essential oil, which are menthol, menthone and 1,8-cineole, with particularly large percentage contents of menthol. However, the compound which contributed to the anti-allergic activity was not known (Sharma et al., 2018). Therefore, this provides a clue for further findings on the possible anti-allergic compound in future. In the work of Lee et al. (2006), it was proposed that the aqueous extract of *Mosla dianthera* exhibited anti-allergic effects through an *in vivo* model. When the mice were sensitized with compound 48/80 and anti-DNP IgE, intraperitoneal pretreatment of 1–1,000 mg/kg of aqueous extract resulted in a dose-related reduction in passive cutaneous anaphylaxis (PCA) reaction (Lee et al., 2006). Similar activities were displayed by the aqueous extract of species *Perilla frutescens* (Shin et al., 2000), *Phlomis umbrosa* (Shin et al., 2008), *Salvia plebeia* (Shin and Kim, 2002), *Schizonepeta tenuifolia* (Shin et al., 1999) and *Teucrium japonicum* aqueous extract (Kim et al., 2009). Sridevi et al. (2009) highlighted that the ethanolic extract of *Ocimum sanctum* at 400 mg/kg effectively reduced mortality (41%) due to anaphylactic shock-induced bronchospasm in tested subjects with a significant drop (P < 0.001) in serum IgE level to 25.80 ± 4.85 ng/ml (P < 0.001), as compared to sensitized control (125.06 ± 9.66 ng/ml). These findings confirm that the anti-allergic potential of *O. sanctum* is worthwhile to be further explored.

Over the past two decades, many studies have been conducted on *P. frutescens* species to explore and determine their anti-allergic potential. For example, Makino et al. (2001) isolated rosmarinic acid **(1)** and apigenin 7-*O*-[β-glucuronosyl (2→1) β-glucuronide] **(2)** from *P. frutescens*, which demonstrated anti-allergic activity with potent suppression of PCA reaction in antigen-challenged mice with inhibition of 41% (P < 0.01) and 32% (P < 0.05) respectively. Meanwhile, Ueda et al. (2002) isolated rosmarinic acid **(1)**, caffeic acid and luteolin **(3)** from the leaves of *P. frutescens* and tested them for respective anti-allergic effects with oxazolone-induced ear edema test. Interestingly, only luteolin **(3)** showed an inhibitory effect on oxazolone-induced ear edema at 1 mg, whereas the other compounds did not show any inhibitory activity (Ueda et al., 2002). However, in a continuation of the work by Makino et al. (2003), the results suggested that the anti-allergic titer of rosmarinic acid **(1)** was 8 folds higher than the conventional anti-allergic drug tranilast, where 19 mg/kg of rosmarinic acid **(1)** was sufficient to achieve an equivalent PCA reaction suppression as 150 mg/kg of tranilast. Such a potent anti-allergic effect from rosmarinic acid **(1)** is certainly exciting and worthy to be further studied in the development of anti-allergic agents. In light of the study by Chen et al. (2015), an OVA-induced murine model of allergic asthma was employed. Results demonstrated a promising reduction in serum IgE level in the OVA-sensitized mice with 320 µg of ethanolic extract of *P. frutescens* leaves and hence amelioration of asthmatic symptoms (Chen et al., 2015). A similar reduction outcome was obtained in the study using *Der f* (*Dermatophagoides farinae*) mite-induced atopic dermatitis murine model with oral administration of *P. frutescens* leaves extract (Komatsu et al., 2016). In the latest work by Kamei et al. (2017), a new active principle, 8-hydroxy-5,7-dimethoxyflavanone (PDMF) **(4)** was isolated from the leaves of *P. frutescens* and demonstrated to have a potent suppressive effect on PCA reaction in anti-DNP IgE-stimulated-BALB/c mice. In addition, sneezing frequency, the allergic rhinitis nasal response, was also reduced with 1.5 mg of PDMF **(4)** after the BALB/c mice were challenged with Japanese cedar pollen grains (Kamei et al., 2017). Considering all these evidences together, *P. frutescens* possesses a great potential to be developed as an effective anti-allergic agent as shown in allergic asthma, atopic dermatitis and allergic rhinitis models.

According to Mizushina et al. (2014), the isolated compound carnosic acid **(5)**, from the *Rosmarinus officinalis* leaves, possesses the ability to suppress PCA reaction at a dose of 100 mg/kg in sensitized mice with percentage inhibition of 67.1%. Interestingly, the inhibition caused by carnosic acid **(5)** was greater than that of tranilast, a frequently used anti-allergic drug. In fact, tranilast at 100 mg/kg only inhibited PCA reaction by 23.9% as compared to 67.1% suppression by carnosic acid **(5)**, which was approximately 2.8 folds stronger than tranilast (Mizushina et al., 2014). Hence, it can be assumed that carnosic acid **(5)** has a strong potential to be used as anti-allergic compound. In another account, *Schizonepeta tenuifolia* demonstrated a 46.26% reduction in serum IgE level with a treatment of 1% of *S. tenuifolia* extract with phosphate buffered saline/olive oil (P/O) in proportion of (9:1) mixture in DNCB-induced BALB/c mice (Choi et al., 2013).

*Salvia miltiorrhiza* is a perennial herb which is well known to have tanshinones as principal bioactives. This plant is widely employed as traditional remedy, particularly in TCM (Li et al., 2015). Over the past decade, researchers have conducted studies to investigate its anti-allergic effect. Yang et al. (2008) proposed that a dose-dependent reduction of PCA reaction occurred at 25–100 mg/kg of ethanol extract of *S. miltiorrhiza* leaves (P < 0.001). When the IgE-stimulated rats were fed with 50 mg/kg of extract, the PCA reaction appeared to decrease by approximately 36.4% (Yang et al., 2008). A study conducted by Trinh et al. (2010a) highlighted the potency of the PCA reaction inhibition exhibited by the active principles of *S. miltiorrhiza* in the following manner: tanshinone I **(6)** (59%) > 15,16-dihydrotanshinone I **(7)** (49%) > tanshinone IIA **(8)** (35%) > cryptotanshinone **(9)** (32%). Another species from the same genus, *S. plebeia*, was reported to suppress *Der f*-induced elevated serum IgE level in BALB/c mice of atopic dermatitis model at a concentration of 100 mg/kg of ethanolic extract (Choi et al., 2014). Therefore, this finding suggests that *S. plebeia* could be a good candidate for atopic dermatitis treatment in future.

For more than 2,000 years, Chinese people have recognized the dried root of *Scutellaria baicalensis* as a very valuable medicinal herb and many people have regarded it as the golden herb due to its diverse medicinal uses. It is traditionally known as Huang-Qin and it is now listed officially in the Chinese Pharmacopoeia (Zhao et al., 2016). It is widely employed in TCM as treatment for cardiovascular diseases and bleeding disorders, such as hematemesis, hematuria and metrorrhagia (Yoon et al., 2009; Chen et al., 2013). In recent years, it has started to emerge as potentially possessing anti-allergic properties as many studies were actively carried out to investigate its anti-allergic effect. Lim et al. (2003) highlighted that *S. baicalensis* root contains active constituents that are particularly useful against allergic diseases. Four flavonoids were isolated from the root of *S. baicalensis*, wogonin (WG) **(10)**, ganhuangenin (GHG) **(11)**, wogonoside (WGS) **(12)** and 3,5,7,2’,6’-pentahydroxyl flavanone (PHF) **(13)**. WG **(10)**, GHG **(11)** and WGS **(12)** were found to potently suppress the production of IgE from the concanavalin A (ConA)-stimulated spleen lymphocytes obtained from Sprague–Dawley rats, at 10 and 100 µM, except PHF **(13)**, even when tested with the highest dose (Lim et al., 2003). This outcome could be due to the structural differences with methoxy substitution and their respective positions on the polyphenolic ring (Lim, 2002). Meanwhile, Li et al. (2014) evaluated the efficacy of three different extracts (ethanol, acetone and ethyl acetate extract) of *S. baicalensis* against allergic reactions. Result revealed that ethanol extract showed the most promising outcome among the three extracts. It demonstrated the highest inhibitory activity against mice ear PCA reaction with percentage inhibition of 55.17% at a dose of 1.42 g/kg.With the same dose, the total serum IgE level was recorded at the lowest level (3.23 ± 1.05 IU/ml) as compared to treatment with the other two extracts and was comparable to the positive control, 0.1 g/kg of sodium cromoglycate (3.19 ± 1.14 IU/ml) (Li et al., 2014). In the latest work of Zhou et al. (2016), another active constituent, oroxylin A **(14)**, isolated from *S. baicalensis*, was reported to cause a potent suppression (P < 0.01) in serum IgE levels in OVA-sensitized mice (Zhou et al., 2016). Other than oral administration, the efficacy of topical application on anti-allergic effect was also evaluated. In the study of Kim et al. (2013), the aqueous extract of *S. baicalensis* was topically applied onto DNFB-induced ear swelling and result suggested that the cutaneous reaction significantly reduced (P < 0.05) by 31% with 5% of extract (Kim et al., 2013). In a continuation of the study from the same researchers a few years later, in addition to cutaneous reactions, the serum IgE level in DNCB-induced contact dermatitis was also proven to be suppressed by topical treatment of *S. baicalensis* aqueous extract (Kim et al., 2016). Considering these results, *S. baicalensis* showed to be effective against IgE production and thus to have preventive effects towards allergy. Hence, it is suitable to be further developed as natural anti-allergic agent.

Furthermore, Zhou et al. (2014) demonstrated that 2-isopropyl-5-methylphenol (thymol) **(15)** from *Thymus vulgaris* portrayed a dose-dependent reduction trend in the production of IgE with the pretreatment of 4 mg/kg, 8 and 16 mg/kg of thymol **(15)** in OVA-challenged mice. Among the three concentrations used, 16 mg/kg of thymol **(15)** showed a comparable inhibition (P < 0.01) of OVA-specific IgE with positive control, dexamethasone (Zhou et al., 2014). Likewise, *Vitex rotundifolia* also showed similar inhibitory activity with 43% suppression in serum IgE level with the treatment of *V. rotundifolia*’s phytoconstituent, 1H,8H-pyrano [3,4-c] pyran-l,8-dione (PPY) **(16)** (Lee et al., 2009).

## Inhibition of Mast Cells and Basophils Degranulation

A few species of Lamiaceae have successfully displayed compelling mast cell stabilizing activity. For instance, Vadnere et al. (2007) evaluated the mast cell stabilizing activity of *Clerodendron phlomidis* using *in vivo* murine system. The mast cell stabilization was achieved by using leaf aqueous extract of *C. phlomidis* in tested mice. Results revealed that 100 mg/kg of extract was able to confer protection as high as 73.25% from mast cell degranulation which was almost comparable to the standard drug, disodium cromoglycate, that exhibited a protection of 83.75% (Vadnere et al., 2007). Furthermore, *O. sanctum* leaves were also studied for their mast cell stabilization activity in the research of Choudhary (2010). The leaves were prepared into ethanolic extract and flavonoidal fraction isolated from ethanolic extract. The albino rats were fed orally with 100 and 200 mg/kg of leaf ethanolic extract after sensitization. The results demonstrated that 62.44 ± 3.80% and 67.24 ± 2.94% of mast cell stabilization activity was respectively recorded at 100 and 200 mg/kg of ethanolic extract. Meanwhile, significant inhibition of mast cell degranulation was also seen with 75 and 150 mg/kg of isolated flavonoidal fraction, which marked with 54.62 ± 1.76% and 60.48 ± 2.72% respectively (Choudhary, 2010). In addition to that, Patel and Deshpande (2011) employed an *in vitro* assay to evaluate the inhibitory activity of mast cell degranulation of *Vitex negundo*. The rat mesenteric mast cells were stimulated with compound 48/80 to induce mast cell degranulation. At the end of experiment, the numbers of intact and disrupted mast cells were counted and compared. Result suggested that the number of intact mast cells was more than that of disrupted mast cells after the pretreatment with 500 µg/ml of aqueous sub-fraction of *V. negundo*. The mast cell protection was significant, which marked with a percentage of 80.99 ± 0.7231% (P < 0.001) (Patel and Deshpande, 2011).

In a study conducted by Murata et al. (2010), it was found that *Lycopus lucidus* contains bioactive compounds that contribute to anti-allergic activity through inhibition of hyaluronidase enzyme (Murata et al., 2010). Hyaluronidase is an enzyme that cleaves hyaluronic acid in an extracellular matrix of connective tissue and is well known for being involved in allergic reactions by causing increased capillary permeability (Sakamoto et al., 1980). Inhibition of this enzyme is known to have suppressive effect on mast cell degranulation, which is a hallmark manifestation of allergy (Asada et al., 1997). Therefore, inhibition of hyaluronidase enzyme can thus become one of the targets to prevent the occurrence of allergy. Murata et al. (2010) isolated 22 compounds from dried aerial parts of *L. lucidus*. Amongst the 22 compounds isolated, only six of them were identified to possess hyaluronidase inhibitory activity. Isolated rosmarinic acid **(1)** was previously identified as a good hyaluronidase inhibitor. It was set as the positive control in this study and marked with hyaluronidase inhibition with IC_50_ value of 309 µM. By comparison, the other five constituents were considered as strong hyaluronidase inhibitors with smaller values of IC_50_ as compared to the positive control. The potencies of inhibition of the six phytoconstituents were arranged in a descending manner: clinopodic acid C **(17)** (IC_50_ value: 80.1 µM) > clinopodic acid E **(18)** (IC_50_: 82.8 µM) > lycopic acid A **(19)** (IC_50_: 134 µM) > lycopic acid B **(20)** (IC_50_: 141 µM) and scizotenuin A **(21)** (IC_50_: 241 µM) > rosmarinic acid **(1)** (IC_50_: 309 µM) (Murata et al., 2010). This has provided scientific evidence to support future research on *L. lucidus* for its anti-allergic potential. Similarly, in the work of Ippoushi et al. (2000), it was found that the leaf methanolic extract of *Melissa officinalis*, which is also commonly known as lemon balm, possessed the highest hyaluronidase inhibition among 46 plants tested, achieving as low as only 1.0 ± 0.3% of enzyme activity (Ippoushi et al., 2000). The potency of lemon balm in suppressing hyaluronidase is worthy to be further explored, so that it can become an anti-allergic therapeutic in future. Taken together, these outcomes suggest that these species can be used as potential novel anti-allergic therapeutic agents through mast cell degranulation inhibition.

## Inhibition of Allergic Mediators and Secretory Granules

Following mast cell degranulation, various chemical mediators are released from the mast cells or basophils. One of the most prominent mediators release from mast cells is histamine. Histamine was discovered as a potent vasoactive agent in 1911 by Dale and Laidlaw and recognized as a major contributor to allergic diseases (White, 1990; Xie and He, 2005). Histamine participates in both early and late phase allergic reactions. In the early phase of allergic reactions, the histamine released from degranulated mast cells and basophils triggers an array of acute allergic symptoms which can be seen within minutes. Some of the examples of allergic symptoms are increased vascular permeability causing redness, swelling, itchiness and pain, bronchoconstriction, anaphylaxis, etc. (Jutel et al., 2009). The onset of immediate allergic reactions is followed by the late phase responses (LAR) which contribute to more sustained inflammation. During the LARs, histamine acts as the chemoattractant to effector cells, notably T_H_2 lymphocytes, eosinophils, and basophils for recruitment to the inflammatory site and hence is implicated in the pathogenesis of late phase chronic allergic inflammatory reactions (He et al., 1997; Galli et al., 2008).

In addition, β-hexosaminidase is another popular biomarker used to evaluate mast cell degranulation in many the allergy studies (Kuehn et al., 2010). Similar to histamine, it is produced and stored as secretory granules within mast cells and basophils. However, unlike histamine, it does not have any significant involvement or contribution to allergic reactions. Instead, β-hexosaminidase was reported to have the ability to confer host defense against bacterial infection (Fukuishi et al., 2014). Nevertheless, it is widely employed as a mast cell degranulation marker because it is released together with histamine during the degranulation process (Cota et al., 2012; Huang et al., 2016). As compared to histamine, the release of β-hexosaminidase is slower and persists for a longer time. This makes β-hexosaminidase a better indicator for mast cell degranulation detection than histamine (Huang et al., 2016). Apart from histamine and β-hexosaminidase, mast cells also contain other mediators like eicosanoids, such as prostaglandin D_2_ and leukotrienes C_4_, chemotactic factors and immunoregulatory cytokines (Xiang et al., 2001; Parikh et al., 2003). During degranulation, these substances are also released from mast cells and basophils through exocytosis. Therefore, they are also used as biomarkers of mast cell degranulation in the allergy studies.

Anti-allergic activity of plants can be evaluated through the potency of the plant in suppressing the mediators, secretory granules or any functional changes induced by the mediators as aforementioned. In a study by Vadnere et al. (2007), a significant inhibition (P < 0.05) in histamine-induced tracheal contraction was seen at concentrations of 4 and 10 mg/ml of leaf aqueous extract of *Clerodendron phlomidis* on isolated goat tracheal chain. At the same time, intraperitoneally administered leaf aqueous extract of *C. phlomidis* at concentrations of 100 mg/kg showed a potent reduction (P < 0.05) in histamine-induced vascular leakage in sensitized murine model (Vadnere et al., 2007). Lee et al. (2011) explored the possibility of *C. trichotomum* on anti-asthmatic potential employing *in vivo* guinea pig model. A phenylpropanoid glycoside, acteoside **(22)** was isolated from the ethyl acetate fraction from *C. trichotomum* leaves. At dose 25 mg/kg, specific airway resistance (sRaw) was significantly (P < 0.05) inhibited in immediate phase response (IAR) and LAR in ovalbumin-challenged guinea pigs by 32.14% and 55.88% respectively. The result seemed promising as it was superior to positive controls, 5 mg/kg dexamethasone (55.88%) and 10 mg/kg disodium cromoglycate (52.94%) in LAR. Meanwhile, concentration of 50 mg/kg of acteoside **(22)** was significantly (P < 0.05) against the histamine release and phospholipase A_2_ (PLA_2_) activity in BALF in which the histamine content was marked with 26.40 ± 1.96% and PLA_2_ activity recorded with only 28.08 ± 2.05% (Lee et al., 2011b).

Park et al. (2010) investigated the anti-allergic potential of *Clinopodium gracile* using both *in vivo* and *in vitro* studies. In the *in vivo* study, the mice were treated with compound 48/80 to induce systemic anaphylaxis after the pretreatment with intraperitoneal administration of aqueous extract of *C. gracile* with concentrations ranging from 1–100 mg/kg. The mortality of mice due to anaphylactic shock was assessed. Results revealed that the systemic anaphylactic death event was reduced concentration-dependently, in which the doses of 50 mg/kg and above were identified as the effective doses that prevented the mice from fatal anaphylactic shock. The reduction of systemic anaphylaxis corresponded to the reduced serum histamine. On the other hand, aqueous extract of *C. gracile* was also shown to significantly (P < 0.05) suppress the release of histamine in *in vitro* RPMC and HMC-1 cells assays. The suppressive effect occurred in a dose-dependent manner with the concentrations ranging from 1–100 µg/ml. As aforementioned, calcium influx is pivotal in the releasing of secretory granules and mediators from mast cell degranulation. Therefore, a reduction of intracellular calcium level can result in the inhibition of chemical mediators such as histamine and β-hexosaminidase release (Oka et al., 2005). In the same study, intracellular calcium level was also evaluated in HMC-1 cell line. Results showed that pretreatment with aqueous extract of *C. gracile* caused a suppression in intracellular calcium level induced by PMACI. Hence, this finding is supported by the theory which suggests the involvement of intracellular calcium in the inhibition of histamine release from mast cells. In addition, mast cell-mediated hypersensitivity also occurs with the involvement of NF-κB and inflammatory cytokines. NF-κB activation is essential to regulate downstream pro-inflammatory cytokine expression, such as TNF-α and IL-6 which play a critical role in initiating and sustaining the allergic inflammatory responses (Blackwell and Christman, 1997). Therefore, the attenuation of NF-κB activation causes a reduction in downstream inflammatory cytokines gene expression and hence produces suppressive effects in allergic inflammation. NF-κB-dependent transcriptional activity was evaluated through luciferase activity assay. Results revealed that treatment with aqueous extract significantly (P < 0.05) inhibited the activation of NF-κB and its downstream cytokine expression (TNF-α and IL-6) at doses of 1 and 10 µg/ml. These findings provide evidence that *C. gracile* is has the potential to be developed as an anti-allergic agent in the future, given its potential in reducing allergic inflammation (Park et al., 2010). Similarly, aqueous extracts of *Dracocephalum argunense* (Kim and Shin, 2006; Kim et al., 2006), *Elsholtzia ciliata* (Kim et al., 2011), *Isodon japonicus* (Kim et al., 2004; Shin et al., 2004), *Lycopus lucidus* (Shin et al., 2005), *Mentha arvensis* (Shin, 2003), *Mosla chinensis* (Kim et al., 2012), *M. dianthera* (Lee et al., 2006), *Perilla frutescens* (Shin et al., 2000), *Phlomis umbrosa* (Shin et al., 2008), *Pogostemon cablin* (Yoon et al., 2016), *Prunella vulgaris* (Shin et al., 2001), *Salvia plebeia* (Shin and Kim, 2002), *Schizonepeta tenuifolia* (Shin et al., 1999), *Stachys riederi* (Shin, 2004), *Teucrium japonicum* (Kim et al., 2009) and *Vitex rotundifolia* (Shin et al., 2000) also exhibited similar outcomes as portrayed in the experiment conducted using *C. gracile*.

In another account, Inoue et al. (2001) prepared four different extracts and separated fractions (50% ethanolic extract, water eluate, 50% ethanolic eluate and ethanolic eluate) from *M. piperita* leaves to investigate their anti-allergic potential on allergic rhinitis. It was found that 50% ethanolic eluate exhibited the most potent inhibition to histamine release, with an IC_50_ value of 2.55 µg/ml and exerted its antagonizing effect on nasal responses at doses of 300 and 1,000 mg/kg in antigen-challenged rats (Inoue et al., 2001). In the continuation of their previous work, Inoue et al. (2002) focused on the 50% ethanolic eluate to isolate the active compounds responsible for the anti-allergic effect. Following the isolation of the compounds, a total of eight chemical constituents were considered. However, among the eight compounds, luteolin-7-*O*-rutinoside **(31)** was found to be the most effective in suppressing histamine release from compound 48/80-induced RPMC with IC_50_ value of 21.9 µM. The nasal responses were reduced at doses of 100 and 300 mg/kg of luteolin-7-*O*-rutinoside **(31)** (Inoue et al., 2002). Recently, Sato and Tamura (2015) evaluated anti-allergic activity of *M. piperita* leaves using β-hexosaminidase assay. Five major flavonoids components, 5,6,4’-trihydroxy-7,8-dimethoxyflavone **(32)**, 5,6,4’-trihydroxy-7,8,3’-trimethoxyflavone **(33)**, 5,6-dihydroxy-7,3’,4’-trimethoxyflavone **(34)**, 5,6-dihydroxy-7,8,3’,4’-tetramethoxyflavone **(35)** and 5,6-dihydroxy-7,8,4’-trimethoxyflavone **(36)** isolated from leaves dichloromethane extract were all shown to possess potent anti-allergic activity. However, 5,6,4’-trihydroxy-7,8,3’-trimethoxyflavone **(33)** and 5,6-dihydroxy-7,8,3’,4’-tetramethoxyflavone **(35)** proved to have the strongest inhibitory activity on β-hexosaminidase release from RBL-2H3 cells, which were recorded with IC_50_ values of 2.4 and 3.0 µM respectively. Their safety profiles also seemed promising as they had relatively lower cytotoxicity than another typical natural anti-allergic substance, luteolin **(3)** (Sato and Tamura, 2015). Therefore, this might indicate that 5,6,4’-trihydroxy-7,8,3’-trimethoxyflavone **(33)** and 5,6-dihydroxy-7,8,3’,4’-tetramethoxyflavone **(35)** can be potentially developed into safe and effective anti-allergic agents. Another species from the same genus, *M. spicata*, was shown to have similar activity with its flavones, 5,6,4’-trihydroxy-7,8,3’-trimethoxyflavone **(33)** and 5,6-dihydroxy-7,8,3’,4’-tetramethoxyflavone **(35)** and aliphatic glycoside, (3R)-1-octan-3-yl β-D-glucopyranoside **(37)**. The potency of inhibitory activities was arranged in a descending manner: 5,6,4’-trihydroxy-7,8,3’-trimethoxyflavone **(33)** with IC_50_ of 6.4 µM > 5,6-dihydroxy-7,8,3’,4’-tetramethoxyflavone **(35)** with IC_50_ of 56 µM > (3R)-1-octan-3-yl β-D-glucopyranoside **(37)** with IC_50_ of 560 µM. Although (3R)-1-octan-3-yl β-D-glucopyranoside **(37)** showed the weakest activity, it was however a great discovery as this was the first time an anti-histaminic activity from an aliphatic glycoside was recorded (Yamamura et al., 1998).

In 2007, Cariddi et al. investigated the anti-allergic activity of *Minthostachys verticillata*. The experiment was conducted using basophils from allergic patients pretreated with essential oil extracted from the stems and leaves of *M. verticillata* and followed by β-hexosaminidase assay. Results suggested that the essential oil showed a promising suppressive effect on β-hexosaminidase release with percentage inhibition ranging from 32.15% to 39.72%, which was comparable to dexamethasone (39.75%) and theophylline (41.63%) (Cariddi et al., 2007). A few years later, Cariddi et al. (2011) carried out an in-depth study on the components of the essential oil extracted from *M. verticillata* to identify the constituent responsible for anti-allergic effect. Limonene **(38)**, pulegone **(39)** and menthone **(40)** were found to be present in the essential oil. Results suggested that limonene **(38)** appeared to be the most effective compound in the inhibition of β-hexosaminidase release from human basophils as compared to the other two compounds, pulegone **(39)** and menthone **(40)**. It was shown that 20 µg/ml of limonene **(38)** was able to achieve inhibitory effect while the other two compounds both required a higher concentration of 40 µg/ml to reach the desired inhibition. The inhibitory effect could be achieved at a lower concentration (10 µg/ml) when the compounds were used in combination (Cariddi et al., 2011). This can be explained with the synergistic effect of the three compounds.

Jeon et al. (2014) highlighted that luteolin **(3)** isolated from the leaves of *P. frutescens* potently (P < 0.05 to P < 0.001) suppressed histamine release from compound 48/80-stimulated RPMC at 5, 10 and 20 µM in a dose-related manner. The expression and production of TNF-α and IL-1β were significantly (P < 0.05 to P < 0.001) reduced in PMACI-stimulated HMC-1 cells, with inhibition rates of 31.9%–76.8% and 27.3%–81.2% respectively, at the range of 5–20 µM of luteolin **(3)** (Jeon et al., 2014). Likewise, Ueda et al. (2002) also reported a similar activity on the TNF-α production with a dose range of 1–1,000 µg of luteolin **(3)** in sensitized mice. A the same time, *in vivo* studies conducted on ICR mice demonstrated that compound 48/80- or serotonin-induced scratching behavior and vascular permeability were dose-dependently decreased at concentrations of 5, 10 and 20 mg/kg of luteolin **(3)** (Ueda et al., 2002). According to a study conducted by Heo et al. (2011) on the anti-atopic effect of *P. frutescens*, the aqueous fraction demonstrated a promising outcome whereby the DNFB-sensitized mice experienced a 35% reduction in ear swelling symptoms with the administration of 100 µg/ml. In light of the study carried out by Zhu et al. (2014), rosmarinic acid methyl ester **(41)** was found to be able to produce a stronger β-hexosaminidase release inhibition than that of rosmarinic acid **(1)** extract prepared from *P. frutescens* leaves using supramolecular technique, in which the IC_50_ values were marked with 9.9 ± 0.8 and 52.9 ± 6.7 µg/ml respectively. Furthermore, Kamei et al. (2017) suggested that PDMF **(4)** significantly (P < 0.05) inhibited histamine release from RBL-2H3 cells with an IC_50_ value of 68.5 µM which was much more potent than other polyphenols. Hence, this finding deduced that PDMF **(4)** is a newly emerged potent anti-allergic component.

In light of a study by Ryu et al. (2000), 2α,3α-dihydroxyurs-12-en-28-oic acid **(42)** isolated from the methanolic extract of *Prunella vulgaris* possessed a significant (P < 0.01) inhibitory activity on β-hexosaminidase release from RBL-2H3 cells with IC_50_ of 57 µM. Another histamine release assay conducted recently by Choia et al. (2016) intended to provide more evidence on the anti-allergic effect of *P. vulgaris*. The results suggested that three triterpenoids isolated from the spike of *P. vulgaris* exhibited significant inhibitory effects on histamine release from HMC-1 cells. The active principles represented by β-amyrin **(43)**, 2α,3α,23-trihydroxyursa-12,20(30)-dien-28-oic acid **(44)** and euscaphic acid **(45)** demonstrated a percentage inhibition of 46.7%, 57.9% and 54.2% respectively. It is noteworthy that 2α,3α,23-trihydroxyursa-12,20(30)-dien-28-oic acid **(44)** was isolated for the first time and possessed promising activity against histamine release (Choia et al., 2016). Putting all the evidence together, *P. vulgaris* is experimentally proven to have high potential to be developed into efficacious anti-allergic agent. On the other hand, Mizushina et al. (2014) highlighted that carnosic acid **(5)** from *Rosmarinus officinalis* leaves significantly inhibited the release of β-hexosaminidase from RBL-2H3 cells at 10 µM.

Likewise, Choi and Kim (2004) and Ryu et al. (1999) reported that 15,16-dihydrotanshinone-I **(7)** and cryptotanshinone **(9)** isolated from the root of *S. miltiorrhiza* showed to have IC_50_ values of 16 ± 2.4 and 36 ± 3.7 µM on β-hexosaminidase release. Additionally, the ethanolic extract of *S. miltiorrhiza* also inhibited other chemical mediators, such as leukotriene C_4_ with a IC_50_ value of 2.6 µg/ml, COX-1 and COX-2-dependent prostaglandin D_4_ with IC_50_ values of 3.96 and 21.54 µg/ml respectively (Yang et al., 2008). With the aid of findings from Yang et al. (2008), Li et al. (2015) successfully identified 15,16-dihydrotanshinone-I **(7)** as the compound responsible for the inhibition of intracellular calcium level, hence suppressing the downstream release of leukotriene C_4_ and prostaglandin D_4_ from activated bone marrow-derived mast cells (BMMC). The inhibition percentage achieved was as high as 90% with a dose of 20 µM of 15,16-dihydrotanshinone-I **(7)** (Li et al., 2015).

In addition, the four active constituents isolated from *Scutellaria baicalensis* root, wogonin (WG) **(10)**, ganhuangenin (GHG) **(11)**, wogonoside (WGS) **(12)**, 3,5,7,2’,6’-pentahydroxyflavanone (PHF) **(13)**, all showed a significant suppression of histamine (P < 0.01) and leukotriene B_4_ (P < 0.01) release from A23817-induced rat peritoneal exudate cells (PEC) at a concentration of 100 µM (Lim, 2003). Moreover, Kim et al. (2010) reported that baicalin **(46)** isolated from the roots of *S. baicalensis* possessed a significant (P < 0.001) inhibitory activity of histamine and leukotriene release from OVA-sensitized guinea pig lung mast cells at 10, 30 and 60 µg. The histamine inhibition rates recorded with 47.1%, 59.4% and 61.5% while leukotriene production remarkably suppressed by 37.9%, 47.3% and 50.4% with the pretreatment of the three different doses of baicalin **(46)**. Interestingly, studies showed that the standardized extract of baicalin **(46)** at high dose produced a greater inhibition on both histamine and leukotriene production as compared to pure baicalin **(46)**. Results revealed that 70.4% of histamine suppression and 78% of leukotriene inhibition (P < 0.001) were found at high dose (60 µg) of standardized extract. However, the percentage inhibitions produced by low and medium dose standardized extract treatments were smaller than that of pure baicalin **(46)** (Kim et al., 2010). In a very interesting study conducted by (Trinh et al., 2010b), baicalin **(46)** isolated from rhizome of *S. baicalensis* was metabolized into baicalein **(47)** and oroxylin A **(14)** followed by oral administration. All three compounds possessed inhibitory activity against histamine-induced scratching behavior and vascular permeability in *in vivo* ICR mice model at doses of 20 and 50 mg/kg. However, the metabolite, oroxylin A **(14)** showed the most potent inhibition instead of its parent compound, baicalin **(46)**. In the study of Li et al. (2014), it was suggested that ethanol extract potently inhibited (P < 0.01) 4-AP-induced allergic skin pruritus, histamine-induced mice paw swelling and cutaneous anaphylactic-ear swelling at 1.42 g/kg. For instance, ear swelling inhibition produced by 1.42 g/kg of ethanol extract (47.10%) was superior to that of the positive control, 0.1 g/kg of cromolyn sodium (32.43%) (Li et al., 2014). In a food allergy study, 25 mg/kg of ethanol extract of *S. baicalensis* conferred 60% protection to food allergy anaphylactic death in OVA-sensitized mice (Shin et al., 2014b). Henceforth, these promising experimental outcomes make the anti-allergic potential of *S. baicalensis* worthwhile to be further explored.

## Regulation of T Cell Responses

It has been long recognized that allergic sensitization is led by lymphocytes (Zhang et al., 2014). There are various types of T lymphocytes, such as CD4^+^, CD8^+^, and natural killer T cells, in which each population produces response to allergens with different capacities. Among the different types of T lymphocytes, CD4^+^ T cells are predominantly implicated in the pathogenesis of allergy. With the expression of major histocompatibility complex (MHC) class II molecules and allergen specific T-cell receptors (TCR), CD4^+^ T cells are able to recognize peptide antigen presented by antigen-presenting cells (APCs). Antigen recognition is then led to the activation of downstream immune responses, such as allergic inflammatory cascade (Kallinich et al., 2007; Woodfolk, 2007).

Naive CD4^+^ T cells can be differentiated towards T_H_1, T_H_2, T_H_17 and Treg phenotypes (Zhang et al., 2014). T_H_2 subsets are particularly renowned as the major contributors to the immunopathology of allergy. Late-phase allergic responses are provoked by the persistent existence of allergens, leading to T-cell activation (Palomares et al., 2010). Activated T_H_2 lymphocytes play a critical role in the production of T_H_2 cytokines (IL-4, IL-5, IL-9 and IL-13) (Deo et al., 2010; Ozdemir et al., 2010). IL-4 and IL-13 are crucial to the development of T_H_2 cells and induction of IgE isotype switching from B cells, which are the major risk factor for the development of allergic asthma (Steinke and Borish, 2001; Woodfolk, 2007). IL-5 mainly mediates eosinophil recruitment and increases eosinophil survival while IL-9 stimulates mast cells and basophils (Woodfolk, 2007; Ozdemir et al., 2010). In contrast, T_H_1 cells differentiation occurs in response to microbial activation of APC under the influence of IL-12 (Deo et al., 2010). These cells orchestrate the production of IL-2, IFN-γ and TNF-β (Romagnani, 2004). T_H_1 cytokines tend to produce pro-inflammatory responses which are important in killing phagocytosed microbes and perpetuating autoimmune responses (Berger, 2000). Recent evidence demonstrate that minimal microbial exposure in early life causes the T_H_1/T_H_2 balance in the immune system to skew towards the pre-allergic T_H_2 response (Berger, 2000; Deo et al., 2010).

Recently, Treg cells emerged as the key component in the sensitization stage of allergy (Zhang et al., 2014). The transcriptional factor, FOXP_3_ serves as a lineage specification factor of Treg cells which is required for the differentiation of Treg cells (Rudensky, 2011; Noval Rivas and Chatila, 2016). FOXP_3,_ which is dominantly expressed by Tregs, inhibits T_H_2 cells activation, thus reducing the production of T_H_2 cytokines, which is essential during the effector phase of allergic reactions (Albano et al., 2013). Treg cells also suppress allergic inflammation through direct action on mast cells, basophils and eosinophils (Palomares et al., 2010; Noval Rivas and Chatila, 2016). It has been shown that constitutive FOXP^+^ Treg controls the symptomatic phase of mast cell activation and IgE-dependent anaphylaxis in mice (Kanjarawi et al., 2013). Apart from that, T_H_17 cells and their corresponding cytokine, IL-17, are also highlighted for their involvement in the progression of T_H_2-mediated allergic disorders (Oboki et al., 2008). A balance between T_H_17 and Tregs is essential for immune homeostasis. Excessive or exaggerated T_H_17 function and elevated T_H_17 cells together with a defect in Treg function or reduction in Treg population lead to the development of allergic disorders, like allergic asthma and rhinitis. Restoring the balance between T_H_17 and Treg can promote the resolution of allergic disorders, such as allergic inflammation seen in allergic asthma (Albano et al., 2013).

Natural compounds with the ability to regulate T cell responses have a great potential in the development of novel anti-allergic therapeutic agents. In this review, there are several plants described to have compelling effects on the down-regulation of T_H_2 cytokines, re-establishment of T_H_1/T_H_2 balance, T_H_17 inhibition and promotion of Treg cells functions. For example, 100 mg/kg of ethanolic extract of *Mentha haplocalyx* was reported to exhibit significant (P < 0.05) inhibition on the expression and production T_H_2 cytokines (IL-4 and IL-5) in BALF (Lee et al., 2011a). Besides, Costa et al. (2012) evaluated the effectiveness of *Ocimum gratissimum* in alleviating allergic asthma. Results revealed that *Blomia tropicalis* mite-immunized and challenged mice receiving 100 mg/kg of *O. gratissimum* methanolic extract had a significant reduction (P < 0.05) in IL-4 level in BALF in relation to those of the untreated group (Costa et al., 2012). In addition, Trinh et al. (2010a) stated that tanshinone I **(6)**, 15,16-dihydrotanshinone I **(7)**, tanshinone IIA **(8)** isolated from *Salvia miltiorrhiza* possessed inhibitory activity on IL-4 and TNF-α expression in IgE-antigen complex-stimulated RBL-2H3 cells at a dose of 50 µM. The ability of *S. miltiorrhiza* in inhibiting IgE-switching cytokine, IL-4 and pro-inflammatory cytokine, TNF-α is thought to be a spring for allergic reactions improvement (Trinh et al., 2010a). Another species from the same genus, *S. plebeia*, was reported to reduce symptoms of atopic dermatitis through regulation of T cells responses (Choi et al., 2014). At a concentration of 100 mg/kg of ethanol extract of *S. plebeia*, the expression of T_H_1, T_H_2 and T_H_17 cytokines was significantly reduced (P < 0.05) in *Der-f*-induced atopic dermatitis-like skin lesions (Choi et al., 2014). On the other hand, *Schizonepeta tenuifolia* demonstrated a significant reduction (P < 0.05) of IFN-γ, IL-4 and IL-13 in IgE-induced allergic model of RBL-2H3 cells with 100 µg/ml of aqueous extract. In the same study, *S. tenuifolia* aqueous extract showed no cytotoxicity even at a higher concentration up to 1,000 µg/ml (Lin et al., 2018). However, a more thorough and detailed toxicological investigation is required to develop a more evidence-based safety profile.

In another account, *Perilla frutescens* also suppressed T_H_2 responses which then contributed to anti-allergic effect (Sanbongi et al., 2004). Results demonstrated that the expression and production of T_H_2 cytokines (IL-4 and IL-5) were potently inhibited by daily oral administration of 1.5 mg of rosmarinic acid **(1)** from *P. frutescens* (P < 0.05) in *in vivo* house mite-challenged murine model (Sanbongi et al., 2004). Meanwhile, Chen et al. (2015) reported that IL-5 and IL-13 were diminished in OVA-induced allergic asthma murine model with ethanolic extract of *P. frutescens* leaves. Interestingly, aqueous fraction of *P. frutescens* leaves showed its effectiveness in alleviating atopic dermatitis through balancing of T_H_1 and T_H_2 cells (Heo et al., 2011). The effect was achieved by suppressing the release of IL-31, which is a T_H_2 cytokine that promotes allergic symptoms like pruritus and allergic skin disorders (Heo et al., 2011; Meng et al., 2018). At the same time, the T-bet protein expression was augmented with 100 µg/ml of *P. frutescens* aqueous fraction (Heo et al., 2011). T-bet protein expression is essential for T_H_1 cell differentiation (Chatila et al., 2008). Therefore, the combination of suppressed IL-31 and augmented T-bet protein expression resulted in T_H_1/T_H_2 balance and hence alleviation of allergic symptoms. Besides, Komatsu et al. (2016) suggested that the CD4^+^/CD8^+^ ratio in splenic T lymphocytes obtained from *Der f*-induced atopic dermatitis NC/Nga mice was decreased from 1.82 ± 0.32% to 1.50 ± 0.07% after receiving treatment of *Perilla* leaves extract. This suppression is believed to be associated with the T_H_1 and T_H_2 balance (Komatsu et al., 2016). Additionally, Liu et al. (2013) employed an *in vitro* assay to investigate the effect of *P. frutescens* extract on *Der p* 2-challenged human bronchial epithelial cells BEAS-2B cells. Result displayed that the pro-allergic cytokines (IL-4, IL-5, IL-13) and GM-CSF productions were all dose-dependently reduced (P < 0.05) in *Der p* 2-stimulated BEAS-2B cells with treatment of 5–50 µg/ml of methanolic extract of *P. frutescens* leaves (Liu et al., 2013).

In a recent work by Bae et al., 2016, *Scutellaria baicalensis* showed to be effective in ameliorating ovalbumin-induced food allergy murine model through the regulation of Treg cells with its natural flavonoid compound, baicalein **(47)**. Results revealed that baicalein **(47)** was able to increase Treg cells population through induction of CD4^+^ FOXP_3_^+^ T cell differentiation without causing any cytotoxicity at concentrations smaller than 10 µmol/L (Bae et al., 2016). In light of a study by Jung et al. (2017), linoleic acid **(50)** in hexane fraction from ethanol extract of *S. baicalensis* roots was reported to cause a significant suppression (P < 0.05) in the production of pro-allergic cytokines (IL-4, IL-5, IL-10 and IL-13) and enhancement of secretion of T_H_1 cytokines (IFN-γ (P < 0.05) and IL-12 (P < 0.01)). The fact that a fatty acid from a plant can contribute anti-allergic effect through restoration of T_H_1/T_H_2 balance is a new discovery (Jung et al., 2017). A similar outcome was noted in a food allergy murine model conducted by Shin et al. (2014b) with *S. baicalensis* ethanol extract. Interestingly, the ethanol extract also down-regulated the IL-17 level produced by T_H_17 and hence produced effective prevention to food allergy (Shin et al., 2014b). Within the same year, the same group of researcher successfully discovered that the active compounds isolated from *S. baicalensis*, represented by wogonin (WG) **(10)**, baicalin **(46)** and baicalein **(47)**, all inhibited the T_H_2 cytokines (IL-4, IL-5, IL-10 and IL-13) and IFN-γ at dose of 50 µmol/L in an *ex vivo* study. However, only WG **(10)** was able to produce inhibition without affecting cell viability as compared to the other two active compounds (Shin et al., 2014a). This finding suggests that WG **(10)** could be a safer anti-allergic compound at a higher dose, but its toxicity profile should be further investigated with more extensive toxicity testings. Kim et al. (2010) focused on the therapeutic potential of *S. baicalensis* on atopic dermatitis. In the experiment, the male NC/Nga mice were orally fed with aqueous extract of *S. baicalensis* after atopic dermatitis-like skin lesion was conventionally developed in the mice. The feeding process was continued for 6 weeks. After 6 weeks, blood was drawn from the mice and analyzed to measure the level of cytokine release. Interestingly, the results revealed that *S. baicelensis* aqueous extract only exhibited specific inhibition on IL-5 (P < 0.05) at a dose of 50 mg/kg. There was no significant change in the level of IL-4 and IL-10 between *S. baicalensis* treatment group and control group (Kim et al., 2010). However, in the study by Kim et al. (2016), it was proposed that topical application of aqueous extract of *S. baicalensis* onto the DNCB-induced contact dermatitis can significantly (P < 0.05) reduce the level of IL-4 and IFN-γ in BALB/c mice skin cells. Zhou et al. (2016) reported that oroxylin A **(14)** isolated from *S. baicalensis* was effective against allergic asthma with significant inhibition (P < 0.01) of IL-4, IL-5 and IL-13 production at doses of 15, 30 and 60 mg/kg of oroxylin A **(14)**. In conclusion, *S. baicalensis* has good therapeutic potential in allergic diseases, such as atopic dermatitis, food allergy and allergic asthma through regulation of T cell responses.

With the intention to explore the effectiveness of 2-isopropyl-5-methylphenol (thymol) **(15)** isolated from *Thymus vulgaris* in alleviating allergic asthma, Zhou et al. (2014) employed an *in vivo* allergic murine model of asthma. Results revealed that thymol **(15)** effectively reduced the symptoms of allergic asthma through dose-dependent inhibition of IL-4, IL-5 and IL-13 production at 4, 8 and 16 mg/kg in OVA-challenged mice. Particularly, a potent reduction (P < 0.01) of OVA-induced T_H_2 cytokines was recorded at 16 mg/kg of thymol **(15)** (Zhou et al., 2014). Apart from thymol **(15)**, carvacrol **(51)** from *T. vulgaris* was reported to effectively inhibit the production of IL-2, IFN-γ, IL-4 and IL-17 cytokines as well as T-bet expression in the *ex vivo* splenocytes cultures (Gholijani and Amirghofran, 2016). On the contrary, both thymol **(15)** and carvacrol **(51)** led to an increase in the level of IL-10 and TGF-β in mice splenocytes cultures (Gholijani and Amirghofran, 2016). Henceforth, the regulation of T cell responses that was shown by thymol **(15)** and carvacrol **(51)** suggests that these two compounds potentially benefit allergic disorders. Likewise, Lee et al. (2009) also highlighted that the active constituent of *Vitex rotundifolia*, 1H,8H-pyrano [3,4-c] pyran-l,8-dione (PPY) **(16)** potently suppressed the IL-5 production in BALF by 34%. Furthermore, hydro-ethanolic extract prepared from *Zataria multiflora* seeds was reported to restore T_H_1/T_H_2 balance by enhancing the ratio of IFN-γ/IL-4 in *in vivo* and *in vitro* assays (Boskabady et al., 2013). Another study conducted by Kianmehr et al. (2017) further supported the above finding. In addition to enhanced IFN-γ/IL-4 ratio, *Z. multiflora* hydro-ethanolic extract also caused potentiation of T_H_1 and suppression effect on T_H_2 and T_H_17 cells, which led to therapeutic effect on allergic asthma in ovalbumin-sensitized BALB/c mice. Results showed that the number of Treg cells (P < 0.001), T_H_1/T_H_2 ratio (P < 0.001), IFN-γ/IL-4 ratio (P < 0.01), IFN-γ (P < 0.05) and FOXP_3_ (P < 0.001) expression were increased significantly. Meanwhile, T_H_2 and T_H_17 cells (P < 0.01 to P < 0.001), IL-4, IL-17 and TGF-β (P < 0.05 to P < 0.001) expressions were significantly reduced in sensitized mice spleen cells (Kianmehr et al., 2017). In conclusion, *Z. multiflora* is able to produce anti-allergic therapeutic effects on type I hypersensitivities like allergic rhinitis, allergic asthma and urticarial by regulating T cell responses.

## Suppression of Eosinophils Migration

Eosinophils are long-lived circulating granulocytes which play a central role in promoting allergic reactions. They arise and differentiate in bone marrow and are then widely distributed in the blood, lungs, thymus, uterus, adipose tissues, spleen, etc and lastly readily migrate to the allergic sites (Fulkerson and Rothenberg, 2013; Wen and Rothenberg, 2016). They migrate to the target sites with the influence of chemoattractant, eotaxin and particularly, IL-5 for eosinophil proliferation, survival and priming (Wen and Rothenberg, 2016). Unlike mast cells and basophils with extensive expression of high affinity IgE receptors, eosinophils have a minimal expression of FcεRI. Nevertheless, they express a great range of cell surface molecules, such as receptors for IgG and IgA, complement receptors, cytokine receptors, whose aggregation can trigger eosinophils activation and development (Rigoni et al., 2018). Often, the number of eosinophils is greatly elevated in circumstances associated with allergic disorders, such as allergic rhinitis, allergic asthma and atopic dermatitis (Stone et al., 2010). Henceforth, natural products with the ability to suppress eosinophil recruitment or infiltration to allergic sites can be the noteworthy pharmacological therapeutic options for allergic disorders.

Several Lamiaceae plant species have demonstrated their suppressive effects on the eosinophil recruitment. Vadnere et al. (2007) showed that intraperitoneally administration of 100 mg/kg leaf aqueous extract of *Clerodendron phlomidis* produced a potent antagonizing effect towards milk-induced eosinophilia by showing a marked reduction in blood eosinophil count in mice with the value of 13.8 ± 2.4 as compared to the control group (43.1 ± 1.25). This reduction is indicative of the effectiveness of *C. phlomidis* in conferring anti-allergic effect. Another species under the same genus, *C. serratum*, was evaluated by Bhangare et al. (2012) to study the anti-allergic effect of its aqueous extract prepared from root and stem using different concentrations. Results demonstrated that all extracts used possessed inhibitory activity on milk-induced leukocytosis. However, the most potent activity was shown by high dose (260 mg/kg) aqueous root extract as compared to low dose (130 mg/kg) or any doses of aqueous stem extracts. This extract accorded a more significant protection than the standard used, dexamethasone from milk-induced leukocytosis (Bhangare et al., 2012). This finding suggests that *C. serratum* might be a potential candidate for anti-allergic therapy. Moreover, Lee et al. (2011b) reported that acteoside **(22)** isolated from *C. trichotomum* at 25 mg/kg significantly (P < 0.05) reduced total leukocytes in BALF from 31.25 ± 6.12 × 10^5^ to 25.23 ± 4.72 × 10^5^. At 50 mg/kg, 29.70% of eosinophil infiltration suppression was achieved in guinea pigs model (Lee et al., 2011b). Similar outcome was seen with 200 µl/kg of *Mentha arvensis* essential oil (Sharma et al., 2018) and 100 mg/kg of ethanolic extract of *M. haplocalyx* in OVA-challenged mice (Lee et al., 2011a). In an attempt to investigate the effect of *Ocimum gratissimum* in alleviating respiratory allergy, Costa et al. (2012) carried out an experiment through *in vivo* model using male A/J mice. The mice were orally fed with 25, 50 or 100 mg/kg of methanolic leaves extract after subcutaneously injecting *Blomia tropicalis* antigen for respiratory allergy induction. Results revealed that *O. gratissimum* significantly suppressed the eosinophil counts (P < 0.05) in BALF as well as eosinophil peroxidase (P < 0.01) level at a dose of 100 mg/kg. The same concentration of methanolic extract *O. gratissimum* also attenuated mucus hypersecretion in airway, which is one of the features of allergic asthma (Costa et al., 2012). Henceforth, this suggests that *O. gratissimum* is effective against respiratory allergy disorders, like asthma.

*Perilla frutescens* is a Lamiaceae plant species which has a global distribution, but is especially concentrated in Asian countries, like China, Japan, Korea and Vietnam among others. In an effort to explore the isolate from *P. frutescens* with anti-allergic activity on respiratory allergy, Sanbongi et al. (2004) evaluated the isolate from the leaves of *P. frutescens*. Results from this study suggested that the isolated compound, rosmarinic acid **(1)**, can bring about a significant reduction in eosinophil infiltration (P < 0.05), BALF eosinophil counts (P < 0.01) and eotaxin expression (P < 0.05) in the *Der f*-challenged C3H/He mice (Sanbongi et al., 2004). Parallel activity was seen in OVA-sensitized BALB/c mice with its ethanolic extract in the study of Chen et al. (2015). On top of that, in an anti-atopic study carried out by Heo et al. (2011), the immunohistochemistry test showed that the eosinophil count was remarkably reduced by 73.7% and the MMP-9 expression was also decreased significantly (P < 0.05) at a 100 µg/ml of *P. frutescens* aqueous fraction (Heo et al., 2011). *Scutellaria baicalensis* is another well-known Chinese herb which is widely used as an anti-allergic herb (Li et al., 2014). In light of the study of Zhou et al. (2016), it was proposed that oroxylin A **(14)** from *S. baicalensis* was effective to alleviate allergic asthma. Results demonstrated that oroxylin A **(14)** at doses of 15, 30 and 60 mg/kg were able to inhibit eosinophil infiltration in BALF and consequently led to reduced airway hyperresponsivess in OVA-challenged BALB/c mice (Zhou et al., 2016). In the same year, Kim et al. (2016) evaluated the efficacy of topical treatment of aqueous extract of *S. baicalensis* on contact dermatitis. Results revealed that topical application was effective in suppressing leukocytes infiltration and hence ameliorated contact dermatitis symptoms (Kim et al., 2016).

In addition, Zhou et al. (2014) reported that 2-isopropyl-5-methylphenol (thymol) **(15)** from *Thymus vulgaris* produced a dose-dependent reduction of eosinophils infiltration in BALF across concentrations of 4, 8 and 16 mg/kg. A remarkable inhibition (P < 0.01) was noted at a dose of 16 mg/kg which was comparable to 2 mg/kg of dexamethasone standard. Consequently, mucus hypersecretion and goblet cell hyperplasia in lung tissues as well as the airway hyperresponsiveness in OVA-sensitized mice in response to methacholine were drastically reduced (Zhou et al., 2014). In 2011, Patel and Deshpande identified the mast cell stabilization potential of *Vitex negundo*. In 2013, they conducted another experiment on *V. negundo*’s leaves to study their anti-asthmatic activity. They successfully isolated a flavonoid compound, 5-hydroxy-3,6,7,3’,4’-pentamethoxyflavone **(52)** from the leaves and found that this compound potently suppressed eosinophil count in BALF (9.50 ± 1.5044; P < 0.05) in egg albumin-sensitized guinea pigs. This result was remarkable as the inhibition portrayed was comparable to that of dexamethasone control (7.50 ± 0.5014; P < 0.05) at a dose of 200 mg/kg (Patel and Deshpande, 2013). This is indicative of the potential of *V. negundo* in the treatment of allergy diseases like asthma. Meanwhile, Lee et al. (2009) highlighted that 1H,8H-pyrano [3,4-c] pyran-l,8-dione (PPY) **(16)** isolated from *V. rotundifolia* fruits remarkably reduced eosinophil migration and eotaxin production by 48% and 70% respectively at 10 µg/ml in human type II-like epithelial lung cells (A549 cell media). In *in vivo* model, the eosinophil count in BALF was also shown to decrease significantly (P < 0.05) by 82% (Lee et al., 2009). In 2011, Koh et al. discovered another new compound, casticin **(53)**, isolated from the fruits of *V. rotundifolia* which also showed to inhibit 63% of eosinophil infiltration at 10 µg/ml while eotaxin production decreased dose-dependently with 0.1–1 µg/ml of casticin **(53)** (Koh et al., 2011). In conclusion, the species mentioned above have the potential to fill the knowledge gap of anti-allergy with the inhibition of eosinophil functions.

## Toxicology

Undeniably, in the past decades, the medicinal potential of Lamiaceae species has captured the attention and interest of many researchers to conduct extensive exploration on their pharmacological properties. Nonetheless, the toxicological aspects of the use of Lamiaceae species have not been studied in-depth. To date, there are several toxicological studies available on Lamiaceae species. For instance, Qi et al. (2009) carried out a toxicological investigation on wogonin **(10)**, the active constituent of *Scutellaria baicalensis*, through acute toxicity testing and sub-chronic toxicity testing in murine models. In the acute toxicity testing, the mice were intravenously administered with 350, 315, 283.5, 255.5 and 229.64 mg/kg of wogonin **(10)** respectively. The mice were observed for their general behavior for an hour after the administration and intermittently observed for 24 h for signs of toxicity up to 14-day duration. In the first hour after treatment, the mice showed decreased locomotor activity, muscle relaxation, catatonia, loss of body-righting reflex and bradypnoea. However, these symptoms diminished within 2 h after treatment. The LD_50_ value of wogonin **(10)** determined was 286.15 mg/kg. Meanwhile, in the sub-chronic toxicity study, doses of 30, 60 and 120 mg/kg of wogonin **(10)** were administered daily to the rats through intravenous route and the rats were put under observation for 90 days. Results revealed that a dose of 120 mg/kg of wogonin can cause heart injury with a long period of intravenous administration **(10)** (Qi et al., 2009).

In another account, the essential oil of *Thymus vulgaris* was evaluated on its toxicological profile (Fachini-Queiroz et al., 2012). Acute toxicity was assessed by administering single oral doses of 2,000, 3,000 and 4,000 mg/kg of *T. vulgaris* essential oil and monitoring for any signs of toxicity for seven consecutive days. There were no apparent behavioral side effects observed in the study and the median lethal dose, LD_50_, of *T. vulgaris* essential oil determined was 4,000 mg/kg. The relatively high LD_50_ value suggested that *T. vulgaris* essential oil is non-toxic and relatively safe for use (Fachini-Queiroz et al., 2012). Haq et al. (2012) evaluated *Vitex negundo* in an acute neurotoxicity study. Results suggested that no signs of neurotoxicity were seen with doses of 250, 500, 1,000 and 2,000 mg/kg in the tested mice. The LD_50_ value determined was greater than 5,000 mg/kg, which indicates the extract of *V. negundo* has a good safety profile (Haq et al., 2012). In term of behavioral symptoms, symptoms started to show at a dose of 2,000 mg/kg, which presented with abdominal contraction, ataxia, reduced spontaneous activity and reduced alertness (Haq et al., 2012).

In China, people have been widely employing *Salvia miltiorrhiza* as a traditional herb for treating a range of cardiovascular disorders (Wang et al., 2012). Therefore, Wang et al. (2012) designed a toxicological study to assess the acute toxicity and sub-chronic toxicity of *S. miltiorrhiza* aqueous extract (Wang et al., 2012). In the acute toxicity investigation, the rats received two doses of 32 g/kg of aqueous extract of *S. miltiorrhiza* through intravenous administration. The sub-chronic toxicity was evaluated by assessment of hematological and biochemical parameters after administering 1.92, 5.76 and 19.20 g/kg of aqueous extract for 13 weeks. At acute doses, no deaths, weight gain or abnormal behavioral changes were observed. The LD_50_ value was deduced to be greater than 64 g/kg. At the same time, sub-chronic toxicity demonstrated that there was a significant reduction (P < 0.05) in haemoglobin concentration in high dose male rats and a significant decrease (P < 0.05) in platelet count, plateletocrit in low dose female rats. The total bilirubin level was also elevated significantly (P < 0.05) in all doses received by rats. As for histopathological findings, focal inflammation was observed at the injection site and the severity increased dose-dependently. Other vital organs showed no abnormalities (Wang et al., 2012).

Despite the widespread interest for the use of *Perilla frutescens*, there were only few toxicological studies documenting the toxic potential of *P. frutescens* (Yu et al., 2017). In spite of the diverse medicinal uses of *P. frutescens*, it is actually a medicinal plant with toxic potential, given that certain plant parts contain high concentrations of toxic phytochemicals. According to Kerr et al. (1986), a high concentration of perilla ketone was accumulated in the flowers and seed parts of *P. frutescens*. Perilla ketone has been reported as a potent pulmonary toxin associated with atypical interstitial pneumonia (Wilson et al., 1977; Guerry-Force et al., 1988; Muller-Waldeck et al., 2010). Furthermore, the toxicological profile of *Mentha arvensis* leaves ethanolic extract was also assessed. The toxic potential of ethanolic leaves extract of *M. arvensis* was tested using brine shrimp cytotoxicity assay. The extracts were serially diluted to concentrations of 1,000, 250, 125, 100 and 75 µg/ml and each extract was added to the tubes containing *Artemia salina*. The number of survivors in each tube was counted after 24 h from the treatment to determine the lethal concentration of ethanolic extract of *M. arvensis* leaves. Result demonstrated that 100 µg/ml as the LD_50_ dose caused a significant cytotoxic activity against the brine shrimp, *Artemia salina* (do Nascimento et al., 2009).

## Conclusion and Future Perspectives

Undeniably, in recent years, there has been an increase in the global demand for natural products for healthcare supplementation. Lamiaceae species have been well known for their culinary values. Nonetheless, they are also a valuable plant family widely employed as medicinal herbs in traditional practices to treat a wide range of allergic inflammatory conditions, such as allergic skin diseases and allergic asthma. Therefore, this plant family may prove to be a diverse source of natural compounds for the development of novel therapeutic agents for allergic disorders. This review conveys deep insights into the botanical features, distribution, medicinal uses, phytochemistry, pharmacology and toxicological investigations conducted, with a particular focus on the anti-allergic activity of Lamiaceae species. A critical analysis of the relationship between phytoconstituents of Lamiaceae species and corresponding anti-allergic activity was done and documented. Currently available *in vitro*, *in vivo* and *ex vivo* studies have provided evidences to support the traditional uses of Lamiaceae species against allergic disorders. Moreover, after a comprehensive summarization, a total of 53 isolated constituents were identified as natural compounds that contribute to anti-allergic effects. In the present review, numerous compounds demonstrated promising anti-allergic activity, such as flavonoids, flavonoid glycosides, diterpenes and phenolics. Some of these constituents were shown to possess comparable anti-allergic activity to the positive control, including carnosic acid (polyphenol), rosmarinic acid, luteolin, oroxylin A, hispidulin and thymol (phenolic), marrubiin (diterpene) and acteoside (phenylpropanoid glycoside). Nevertheless, in the current review, it was found that many studies were conducted using plant extracts. Although the extracts were proven to exhibit anti-allergic effect, the active constituent responsible for the effect is yet to be identified. Therefore, further studies should be carried out on the plant extracts or fractions which exhibited promising experimental results in order to elucidate the exact active principles responsible for anti-allergic activity. With the availability of the information regarding phytochemicals, other studies such as the standardization of extracts and pharmacological studies as well as toxicological investigations of isolated bioactives can be conducted. Meanwhile, the natural compounds that have been successfully isolated should be further explored and scrutinized for their therapeutic potential to establish a more evidence-based clinical profile. Henceforth, the development of lead molecules for drug discovery can be quicken and accelerated.

## Author Contributions

SLY obtained the literatures and wrote the manuscript, while NZAR and KH edited the manuscript.

## Funding

This study was financially supported by Universiti Kebangsaan Malaysia (UKM) under grant number GUP- 2018-138.

## Conflict of Interest Statement

The authors declare that the research was conducted in the absence of any commercial or financial relationships that could be construed as a potential conflict of interest.
